# Cationic mRNA Lipid Nanoparticles for Ex Vivo NanoCAR‐T Cell Engineering

**DOI:** 10.1002/advs.202521507

**Published:** 2026-03-24

**Authors:** Laure Harinck, Stijn De Munter, Margo De Velder, Joline Ingels, Dominika Berdecka, Ine Lentacker, Winnok H. De Vos, Bart Vandekerckhove, Kevin Braeckmans, Koen Raemdonck

**Affiliations:** ^1^ Laboratory for General Biochemistry and Physical Pharmacy Faculty of Pharmaceutical Sciences Ghent University Ghent Belgium; ^2^ Cancer Research Institute Ghent (CRIG) Ghent Belgium; ^3^ Department of Diagnostic Sciences Faculty of Medicine and Health Ghent University Ghent Belgium; ^4^ GMP Unit Cell & Gene Therapy Ghent University Hospital Ghent Belgium; ^5^ Cell Biology and Histology Lab University of Antwerp Antwerp Belgium; ^6^ Antwerp Centre for Advanced Microscopy Antwerp Belgium

**Keywords:** CAR‐T cells, immunotherapy, lipid nanoparticles, mRNA delivery, non‐viral gene delivery

## Abstract

Lipid nanoparticles (LNPs) are proposed as an attractive non‐viral alternative for mRNA‐based CAR‐T cell engineering. However, the impact of the LNP charge and the composition of the transfection medium remain to be fully explored. Here, charge‐neutral C12‐200 LNPs and cationic DOTAP(C12‐200) LNPs were compared for ex vivo transfection of activated primary human T cells. C12‐200 LNPs achieved efficient transfection with high cell viability in serum‐free medium supplemented with apolipoprotein E. Transfection efficiency correlated with low‐density lipoprotein receptor expression following T cell activation, suggesting receptor‐mediated uptake. In contrast, cationic LNPs demonstrated superior mRNA expression independent of medium composition or T cell activation state, albeit with reduced cytocompatibility. Both LNPs efficiently delivered anti‐CD20 nanoCAR mRNA into primary T cells, enabling potent cytotoxicity against CD20^+^ Raji cells in vitro. Together, our findings demonstrate that LNP charge, selected proteins in the culture medium, and T cell activation collectively play a critical role in ex vivo T cell engineering. Here, cationic LNPs offer the advantage of unspecific charge‐dependent transfection, inducing high mRNA expression independent on T cell activation status or complex cell culture media. These traits offer opportunities to extend expression kinetics, transfect less activated T cell phenotypes, and simplify CAR‐T cell manufacturing.

## Graphical abstract

This study compares charge‑neutral and cationic lipidnanoparticles (LNPs) for ex vivo mRNA delivery to T cells, revealing distinctdependencies on medium composition and T cell activation. Where conventional LNPs benefit from ApoE‐dependent T cell targeting, cationic DOTAP‑modified LNPs mediate unspecific charge‑dependent transfection. The latter reduces medium complexity and could enable transfection across a broader spectrum of T cell phenotypes.

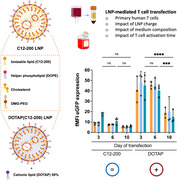

## 1 Introduction

Rapid improvements in chimeric antigen receptor (CAR)‐T cell therapy for hematological malignancies have significantly contributed to establishing immunotherapy as a principal cancer treatment [1, 2]. As of July 2025, seven CAR‐T cell therapies have been approved by the Food and Drug Administration (FDA) for the treatment of B‐cell lymphomas, acute lymphoblastic leukemia (ALL), and multiple myeloma [3, 4, 5, 6, 7]. The most recent FDA‐approved therapy, Aucatzyl (Obecabtagene autoleucel), has been granted conditional marketing authorization at the European Medicines Agency (EMA) for patients with B‐ALL [8]. All these therapies are autologous CAR‐T cell products in which T cells are isolated from the patient, activated, and modified ex vivo to introduce the CAR receptor. The latter enables T cells to specifically recognize and eliminate tumor cells by HLA‐independent targeting of tumor‐associated antigens. Subsequently, the engineered T cells are expanded and re‐infused into the patient in combination with lymphodepleting chemotherapy [9, 10]. Up until today, the approved CAR‐T cell therapies have been produced using lentiviral or gammaretroviral vectors [11]. Such vectors allow for stable integration of the CAR transgene into the T cell genome, leading to long‐term CAR expression [12]. Nevertheless, the use of viral vectors entails important drawbacks such as limited packaging capacity, inconsistent transduction yields, and high manufacturing costs [11, 12, 13]. In addition, GMP production of viral capsids is labor‐intensive and difficult to scale up with long lead times due to limited production capacity, which results in a further increase of production costs and a decrease in accessibility of personalized CAR‐T cell treatment [11, 14].

To address these challenges, non‐viral transfection strategies constitute an attractive alternative [15]. Electroporation, a state‐of‐the‐art physical delivery technology, enables the intracellular delivery of large payloads [16, 17]. However, its use is hampered by significant cytotoxicity and undesired variance in gene expression and T cell functionality [18, 19]. Therefore, more gentle non‐viral transfection methods, including lipid nanoparticles (LNPs), are of current interest for T cell engineering, as they provide several advantages [15, 20]. LNPs allow for a variety of cargoes to be delivered in cells, including mRNA, pDNA, and proteins such as CRISPR‐Cas9 ribonucleoprotein (RNP) complexes, without affecting T cell viability or inducing marked phenotypic alterations [14, 21, 22, 23, 24, 25]. In addition, LNPs represent a robust and easily scalable nucleic acid delivery platform, which allows to produce more cost‐effective therapies compared to viral vectors [11, 26, 27].

The non‐viral introduction of CAR receptors in T cells can be obtained using diverse engineering methods, which include both integrating approaches, such as CRISPR or transposons, and non‐integrating strategies, like plasmid DNA (pDNA) or mRNA encoding for a CAR [14, 28, 29]. Specifically, mRNA allows a transient CAR expression, offering multiple advantages. First, rapid and easy production of mRNA significantly reduces costs compared to viral vectors [30]. Second, due to its transient nature, the risk for on‐target off‐tumor toxicity can be mitigated as the CAR‐T cells will not persist for a prolonged time in the patient [24, 30]. In principle, transient mRNA CAR‐T cells enable repeated dosing strategies that may enhance functionality. Unlike stably engineered lentiviral CAR‐T cells, which become progressively hypofunctional under prolonged tumor microenvironment exposure, freshly dosed transient CAR‐T cells could avoid exhaustion and improve efficacy [31]. On the other hand, this approach would require the availability of sufficient patient‐derived T cells and a significant reduction in current manufacturing costs to ensure feasibility. In addition, LNPs potentially enable co‐delivery of various mRNAs, encoding different receptors, each targeting distinct tumor‐associated antigens. As heterogeneity in tumor tissue remains a formidable challenge, the prospect of simultaneously targeting multiple tumor antigens is a major advantage [30, 31, 32].

Nevertheless, T cells remain notoriously hard‐to‐transfect [33]. Libraries of diverse charge‐neutral LNP compositions, mainly focusing on distinct ionizable lipid designs, have been screened for ex vivo T cell engineering, albeit with varying success [18, 24, 34, 35]. Of note, Cheng et al. developed Selective Organ Targeting (SORT) LNPs, for example, by additionally incorporating a permanently charged cationic lipid, to mediate tissue‐specific RNA delivery in vivo [36]. Positively charged LNPs may offer advantages for ex vivo T cell transfection as well, by enhancing electrostatic interactions with the T cell membrane and facilitating endocytosis. However, such LNP formulations have not been explored for this purpose to date. Together with the LNP surface charge, the composition of the transfection medium may have a profound impact on T cell binding and transfection efficiency. For instance, apolipoprotein E (ApoE) adsorption to the surface of charge‐neutral, but not cationic, LNPs was shown to facilitate low‐density lipoprotein (LDL) receptor‐mediated RNA delivery into hepatocytes following intravenous administration [37, 38, 39, 40]. Likewise, Dilliard et al. demonstrated that LNP charge dictates the preferential adsorption of well‐defined serum proteins in vivo, which influences binding to and transfection of specific cell types [38, 39]. For ex vivo T cell engineering, given the variability and safety concerns associated with serum, the use of chemically defined serum‐free medium supplemented with specific recombinant proteins is favored [41, 42, 43]. It is conceivable that such proteins likewise interact with the LNP surface, which could influence T cell binding. Nevertheless, the interplay between LNP charge and transfection medium composition and its impact on the efficacy of ex vivo T cell engineering remains unclear to date.

It is well‐known that T cells upregulate both the LDL and transferrin receptor upon activation. This study aims to investigate how LNP charge (i.e., LNPs modified with the permanently charged cationic lipid DOTAP) and medium composition (i.e., chemically defined T cell culture medium spiked with ApoE or transferrin) affect cell uptake and mRNA‐based nanoCAR engineering of primary human T cells as a function of time post‐activation. Our data reveals that the LNP charge defines the need for distinct transfection conditions to achieve optimal T cell engineering for the manufacturing of functional nanoCAR‐T cells ex vivo.

## 2 Materials and Methods

### 2.1 Cell Lines, Primary Human T Cells, and Culture Conditions

Jurkat E6.1 cells (ATCC no. TIB‐152, Research resource identification (RRID): CVCL_0367), an immortalized T lymphoblast cell line, were obtained from American Type Culture Collection and cultured in Roswell Park Memorial Institute (RPMI) 1640 medium (Gibco, Thermo Fisher Scientific, Waltham, MA, USA) supplemented with 10% fetal bovine serum (FBS, Biowest, Nuaillé, France), 100 U/mL penicillin, 100 µg/mL streptomycin (P/S, Gibco, Merelbeke, Belgium) and 2 mg/mL L‐glutamine (Sigma–Aldrich, St. Louis, MO, USA). Mycoplasma contamination was assessed using the MycoAlert Mycoplasma detection kit (Lonza, Basel, Switzerland) according to the manufacturer's instructions, and all cell cultures were tested negative. Cell culture concentration was maintained between 1 × 10^5^ and 1 × 10^6^ cells by adding fresh medium every 2 to 3 days, depending on the cell density. Prior to transfection, Jurkat cells were seeded at a concentration of 6 × 10^4^ cells/well in a 96‐well‐plate.

Human buffy coats were obtained with informed consent from the Red Cross Flanders Biobank (Ghent, Belgium). The donor material was used following the guidelines of the Medical Ethical Committee of Ghent University Hospital (Ghent, Belgium). Peripheral blood mononuclear cells (PBMCs) were isolated from buffy coats using density gradient centrifugation with Lymphoprep (Stem Cell Technologies, Vancouver, Canada). After centrifugation, the freshly isolated PBMCs were washed in Dulbecco's Phosphate Buffered Saline (DPBS, Sigma–Aldrich, St. Louis, MO, USA), and the remaining red blood cells were lysed by ACK Lysing buffer (Invitrogen). Human pan CD3^+^ T cells were isolated by magnetic negative selection using the EasySep Human T Cell Isolation Kit (Stem Cell Technologies, Vancouver, Canada) according to the manufacturer's instructions. T cells were seeded at a final concentration of 1 × 10^6^ cells/mL in Iscove's modified Dulbecco's medium (IMDM) GlutaMax (Gibco, Thermo Fisher Scientific, Waltham, MA, USA), supplemented with 10% FBS, 100 U/mL penicillin, and 100 µg/mL streptomycin (complete IMDM, cIMDM). Depending on the performed experiment, T cells were activated using 10 µL/mL per 1 × 10^6^ cells/mL of ImmunoCult Human CD3/CD28/CD2 T Cell Activator (Stem Cell Technologies, Vancouver, Canada) and supplemented with 10 ng/mL IL‐2 (Human Recombinant IL‐2, CHO‐expressed, StemCell, Vancouver, Canada). Stimulated T cells were kept in culture for up to 10 days, and concentration was maintained between 5 × 10^5^ and 1 × 10^6^ cells/mL by adding fresh cIMDM. Before transfection, T cells were seeded at 5 × 10^4^ cells/well in a 96‐well‐plate. Mycoplasma contamination of primary human T cells was assessed using the MycoAlert Mycoplasma detection kit (Lonza, Basel, Switzerland) according to the manufacturer's instructions, and all tested cultures were free of contamination.

### 2.2 mRNA and IVT Production

CleanCap eGFP mRNA with 5‐methoxyuridine (5moU), CleanCap firefly luciferase (Luc) mRNA (5moU) (Trilink Biotechnologies, San Diego, CA), or in vitro transcribed (IVT) anti‐CD20 nanoCAR mRNA was used as specified in different experiments. The mRNA encoding for anti‐CD20 nanoCAR was kindly provided by the lab of Prof. Bart Vandekerckhove (Faculty of Medicine and Health Sciences, Ghent University, Ghent, Belgium). The anti‐CD20 nanoCAR construct was designed with a sequence encoding for CD20 nanobody combined with the CD8α hinge and transmembrane region, followed by the 4_1BBζ and CD3ζ intracellular signaling moieties [44]. The IVT reaction, precipitation, and purification of the anti‐CD20 nanoCAR mRNA were performed as described by Ingels et al. [45]. Here, N1‐methyl‐pseudouridine‐5’‐triphosphate (ThermoFisher Scientific) and CleanCap AG (Trilink Biotechnologies, San Diego, CA) were used as nucleotide modification and capping reagents, respectively.

### 2.3 LNP Formulation and Characterization

DOTAP (1,2‐dioleoyl‐3‐trimethylammonium propane), cholesterol, DOPE (1,2‐dioleoyl‐sn‐glycero‐3‐phosphoethanolamine), DMG‐PEG 2000 (1,2‐dimyristoyl‐rac‐glycero‐3‐methoxypolyethylene glycol‐2000) were purchased from Avanti Polar Lipids (Alabaster, USA). C12‐200 ionizable lipid was obtained from Corden Pharma (Plankstadt, Germany). Alternative ionizable lipids used were SM‐102 and ALC‐0315, purchased from BroadPharm (Cat. No. BP‐25499 and BP‐25498, respectively), and DLin‐MC3‐DMA (Cat. No. HY‐112251, MedChemExpress). LNP formulations were prepared using the microfluidic mixing technique with a NanoAssemblr Spark (Precision Nanosystems, Vancouver, Canada). Briefly, lipid components (ionizable lipid, DOPE, cholesterol, DMG‐PEG) were dissolved in ethanol at molar ratios of 50:10:38.5:1.5. For cell interaction studies, 0.5% to 1.2% DiD dye (1,1'‐dioctadecyl‐3,3,3',3'‐tetramethylindodicarbocyanine, 4‐chlorobenzenesulfonate salt, Invitrogen, Merelbeke, Belgium) was added to the lipid mixture. DOTAP(C12‐200) LNPs contain an additional fifth lipid component with molar ratios of 50:8:23.3:1.3:17.5 (DOTAP:DOPE:cholesterol:DMG‐PEG:C12‐200). The aqueous phase was prepared by dissolving mRNA in sodium acetate (NaOAc) buffer (50 mm, pH 4). For all LNP formulations, the total amount of ionizable lipids was calculated to obtain a nitrogen‐to‐phosphate ratio (N/P) of 6, and the two phases were mixed at a 1:2 volume ratio (EtOH:aqueous). After formulation, LNPs were 1:1 diluted in PBS^−/−^ (w/o Ca^2+^ and Mg^2+^, pH 7.4) and stored at 4°C for a maximum of 2 weeks. A total final lipid concentration of 3.05 and 2.69 mg/mL was obtained for C12‐200 and DOTAP(C12‐200) LNPs, respectively. As quality control, the hydrodynamic diameter and polydispersity index (PDI) were determined using a Malvern Zetasizer nano‐ZS (Malvern Instruments Ltd., Worcestershire, UK) at 25°C. LNPs were diluted 1/50 in HEPES buffer (20 mm, pH 7.4). The ζ‐potential was measured with a Malvern Zetasizer nano‐ZS (Malvern Instruments Ltd., Worcestershire, UK) at 25°C by using a folded capillary zeta cell (DTS1070). mRNA encapsulation efficiency (EE) and final concentration were quantified by the Quant‐iT RiboGreen RNA Assay (Invitrogen, Merelbeke, Belgium) according to manufacturer's protocol.

$$EE\;\;\left(\%\right)=\frac{Total\;mRNA-Free\;mRNA}{Total\;mRNA}\;*100$$

### 2.4 Quantification of LNP‐Mediated mRNA Delivery in Jurkat Cell Line Using Flow Cytometry

Confluent Jurkat cells were counted using Trypan Blue exclusion dye (Merck, Darmstadt, Germany) and resuspended to a concentration of 1.2 × 10^6^ cells/mL in the appropriate cell culture medium, specified for each experiment. The media used were RPMI 1640, X‐VIVO 15 serum‐free hematopoietic cell medium (Lonza, Cat. No. BE02‐060F, Basel, Switzerland) or ImmunoCult‐XF T Cell expansion medium (IM‐XF) (Stem Cell, Cat. No. #10981, Vancouver, Canada). The latter two were spiked with additional Human Transferrin Protein (Acro Biosystems, Cat. No. TRN‐H52H3, His Tag (MALS verified)) or Human Apolipoprotein E3 (Acro Biosystems, Cat. No. APE‐H5246, His Tag) at a concentration of 1 µg/mL (Table S1). Cells were seeded at 6 × 10^4^ cells/well in 50 µL medium in a flat‐bottom 96‐well‐plate. LNPs were diluted in the corresponding cell culture medium (with spiked proteins) and added to the cells at a concentration of 1 ng/µL mRNA based on the final encapsulated concentration determined with the Ribogreen Assay. The cells were incubated for 24 h at 37°C with 5% CO_2_. Flow cytometry was performed to evaluate eGFP expression after 24 h of incubation with the LNPs. Cells were transferred to a U‐bottom 96‐well‐plate, centrifuged, and resuspended in 100 µL flow buffer (PBS ^−/−^ with 1% BSA and 0.1% NaN_3_). Samples were measured using a CytoFLEX flow cytometry plate reader for 96‐well‐plates (Beckman Coulter, Krefeld, Germany) and CytExpert software (Version 2.1). eGFP protein and DiD dye were excited with 488 and 638 nm lasers and detected with 525/40 and 660/20 filters, respectively. FlowJo software (Version 10.8.1, Treestar Inc.) was used to perform data analysis.

### 2.5 Evaluation of LNP‐Mediated mRNA Delivery in Activated Primary Human T Cells Using Flow Cytometry and Confocal Microscopy

Primary human CD3^+^ T cells were isolated from PBMCs, activated, and kept in culture as previously described. On day 3, day 6, and day 10 post‐activation, T cells were collected, counted, and resuspended at a concentration of 1 × 10^6^ cells/mL in IM‐XF spiked with additional Human Transferrin Protein or Human Apolipoprotein E3 (ApoE) at a concentration of 1 µg/mL. Cells were seeded at a cell density of 5 × 10^4^ cells/well in 50 µL of medium in a flat bottom 96‐well‐plate. LNPs containing eGFP or Luc mRNA were diluted to a concentration of 2 ng/µL in IM‐XF with or without additional proteins. The diluted LNPs were added 1:1 to the seeded T cells, providing a final concentration of 1 ng/µL mRNA and the cells were incubated for 24 h at 37°C and 5% CO_2_. After 24 h, detection of eGFP expression was performed as described above via flow cytometry. To correct for autofluorescence, each condition was normalized to the fluorescence of cells treated with Luc mRNA LNPs. For the determination of LNP‐T cell interaction, transfected T cells were visualized with a Nikon A1R HD confocal laser scanning microscope (Nikon Benelux, Belgium) with a 60x water immersion lens (SR plan apo IR 60X WI, NA 1.3, WD 80 µm). In detail, T cells were transfected with LNPs containing DiD lipid dye at a concentration of 1.2 mol%. After 24 h incubation, the cells were stained with 1/2000 dilution of Hoechst 33342 (Invitrogen, Belgium) in PBS^−/−^ for 10 min at 37°C. Subsequently, the cells were washed and resuspended in IM‐XF. 408 and 633 nm laser lines were used to determine eGFP expression and the presence of DiD‐LNPs in T cells. Further processing of the images was achieved using ImageJ software.

### 2.6 Quantification of Cell Viability with CellTiter‐Glo Assay

The CellTiter‐Glo luminescent cell viability assay (Promega, Belgium) was used according to the manufacturer's protocol to determine cell viability after 24 h incubation with LNPs. In this assay, the number of viable cells after LNP treatment can be determined based on quantitation of the ATP present, as an indicator of metabolically active cells. In brief, a volume of CellTiter‐Glo reagent equal to the LNP‐T cell culture was added directly to the cells and shaken on an orbital shaker (120 rpm) at room‐temperature to allow for cell lysis. After 10 min, the cell lysates were transferred to an opaque flat‐bottom 96‐well‐plate, and the plate was incubated for 10 min at room‐temperature to stabilize the luminescent signal. Next, luminescence was measured using a GloMax microplate reader (Promega, Belgium) with a detection wavelength of 350 to 650 nm. To calculate cell viability, the luminescence of LNP‐treated T cells was normalized to non‐treated control samples that were exposed to the same activation protocol.

To determine transfection yield, the cell viability data were combined with transfection efficiency measured by flow cytometry. This parameter represents the percentage of viable transfected cells of the initial cell population. The percentage transfection yield can be calculated by multiplying the percentage of eGFP‐positive cells and the percentage of viable cells:

$$Yield\;\;\left(\%\right)=\frac{transfection\;efficiency\;\left(\%\right)\;x\;cell\;viability\;\left(\%\right)}{100}$$

### 2.7 Analysis of Receptor Expression After T Cell Stimulation

To analyze the receptor expression and activation status of T cells after initial stimulation, cells were stimulated with ImmunoCult CD3/CD28/CD2 activator and kept in culture for up to 10 days. On day 0, 3, 6, and 10 post‐activation cells were stained using the following anti‐human monoclonal antibodies: CD25‐APC (StemCell technologies, Vancouver, Canada), CD71‐APC (Biolegend, USA), and LDLR‐BV421 (BD biosciences). Briefly, cells were centrifuged at 1 × 10^5^ cells/100 µL in a U‐bottom 96‐well‐plate at 500 g for 5 min. Subsequently, the cells were resuspended in PBS^−/−^ containing Human TruStain FcX (Biolegend, USA) at a concentration of 0.5% and incubated for 20 min at room‐temperature. Next, cells were washed in flow buffer (PBS^−/−^ with 1% BSA and 0.1% NaN_3_) and incubated in flow buffer containing the indicated antibodies for 30 min at 4°C. Sytox Green Nucleic Acid Stain (Invitrogen, Belgium) was used to distinguish between live and dead cell populations. After a washing step, cells were resuspended in flow buffer with 0.08% Sytox Green and analyzed with a MACSQuant Analyzer 16 (Miltenyi Biotec, Germany). Data analysis was performed using FlowJo software (Version 10.8.1, Treestar Inc.).

### 2.8 Optimization of Transfection Parameters for mRNA Delivery in Primary Human T Cells

#### 2.8.1 mRNA Dose Titration

To assess the influence of mRNA dose on transfectability and toxicity, the same protocol as described above was used with some minor changes. Briefly, T cells were transfected in both IM‐XF and IM‐XF + ApoE (1 µg/mL). Transfections were performed on day 3 and 6 post‐activation with a varying mRNA concentration of 0.5, 1, 2, and 3 ng/µL mRNA to 5 × 10^4^ T cells/100 µL. After 24 h incubation, eGFP expression was determined using flow cytometry, and cell viability was assessed by CellTiterGlo assay.

#### 2.8.2 Evaluation of mRNA Kinetics in T Cells

The kinetics of mRNA expression after LNP‐mediated delivery was assessed by transfection of activated T cells on day 3. Here, eGFP and Luc mRNA LNPs were added to 5 × 10^4^ cells/100 µL at a concentration of 3 ng/µL mRNA in both IM‐XF and IM‐XF + ApoE (1 µg/mL). Cells were incubated at 37°C and 5% CO_2_. After 24 h, LNPs were removed by centrifugation (4 min at 400 g), and the cells were resuspended in fresh IM‐XF with additional ImmunoCult Human CD3/CD28/CD2 T Cell Activator (1 µL/mL). eGFP expression was evaluated after 24, 48, 72, and 96 h post‐transfection via flow cytometry. Luc mRNA LNPs were used to correct for autofluorescence during data analysis.

### 2.9 nanoCAR Expression and Determination of Kinetics

Activated CD3^+^ human T cells were transfected with C12‐200 or DOTAP(C12‐200) LNPs containing anti‐CD20 nanoCAR mRNA at a concentration of 3 ng/µL or 1 ng/µL, respectively. Transfections were performed with 5 × 10^4^ cells/100 µL, and cells were resuspended in either IM‐XF or IM‐XF + ApoE (1 µg/mL). The LNP‐treated cells were incubated at 37°C and 5% CO_2_, and LNPs were removed after 24 h. NanoCAR expression kinetics were determined after 24, 48, 72, and 96 h post‐transfection via flow cytometry. For analysis of nanoCAR expression on the surface, T cells were stained with an iFluor 488‐conjugated MonoRab Rabbit Anti‐Camelid VHH (Genscript by Bio‐Connect, Huissen, The Netherlands). Briefly, cells were transferred to a U‐bottom 96‐well‐plate and incubated with 0.5 µL Human Trustain FcX (Biolegend, USA) in 100 µL PBS^−/−^ for 20 min at room‐temperature. Next, cells were washed in flow buffer and centrifuged. After removal of the supernatant, staining of the CAR receptors was performed in flow buffer using an antibody concentration of 0.5 µL per 1 × 10^5^ cells/100 µL, and the cells were incubated for 30 min at 4°C. 7‐AAD Viability Staining solution (Biolegend, USA) was added to the flow buffer before analysis to distinguish between live and dead cell populations. Samples were measured on a CytoFLEX flow cytometer (Beckman Coulter, Krefeld, Germany) and CytExpert software (Version 2.1). 7‐AAD Viability dye and iFluor‐488 Anti‐Camelid VHH were excited with a 488 nm laser and detected with 690/50 and 525/40 filters, respectively. Data analysis was performed using FlowJo software (Version 10.8.1, Treestar Inc.).

### 2.10 Tumor Cell Killing Efficiency of Anti‐CD20 nanoCAR‐T Cells

To evaluate the functionality of the transiently engineered anti‐CD20 nanoCAR‐T cells, an in vitro cytotoxicity assay was performed using luciferase‐expressing CD20^+^ Raji cells (Burkitts lymphoma). In detail, Raji cells (target cells, T) were plated in an opaque 96‐well‐plate at a density of 5 × 10^3^ cells/well in cIMDM. nanoCAR‐T cells (effector cells, E) were cocultured at different ratios E:T (i.e., 40:1, 20:1, 10:1, 5:1, 1:1). The total transfected T cell population, consisting of engineered as well as non‐transfected T cells, was used, resulting in identical total cell numbers for each specific E:T ratio. As a positive control, Raji cells incubated with 2% Triton X‐100 were used to induce cell death. The plates were centrifuged and incubated at 37°C and 5% CO_2_. After 24 h, 50 µL of D‐luciferin (0.3 µg/µL in cIMDM) (IVISbrite D‐Luciferin Potassium Salt Bioluminescent Substrate, Revvity, Belgium) was added to the cells and incubated for 10 min at room‐temperature. Subsequently, the luminescent signal was determined using the GloMax Discover microplate reader (Promega, Belgium). The percentage of lysis was calculated using the following formula, with background described as positive control (Raji + Triton):

$$\begin{array}{ccc} & & \%\;Lysis=\\ & & \left(1-\frac{\left(Luminescence\;coculture\;samples-background\right)}{\left(Luminescence\;Raji\;cells-background\right)}\right)*100 \end{array}$$

### 2.11 Statistical Analysis

Experiments were typically performed in technical triplicates with three or more biological repeats or independent donors. All data are presented as mean ± standard deviation (SD) unless otherwise stated. Statistical analyses were performed using GraphPad Prism v9.0.0 (La Jolla, USA). Two‐way ANOVA with Tukey's multiple comparison test was used to compare transfection efficiencies across cell culture media in both Jurkat cells and primary human T cells. The same test was applied to assess statistical differences in mRNA dose titration experiments. Additional statistical tests are specified in the corresponding figure legends. A *p* value <0.05 was considered statistically significant (ns *p* > 0.05; ^*^ *p* < 0.05; ^**^ *p* < 0.01; ^***^ *p* < 0.001; ^****^ *p* < 0.0001).

## 3 Results

### 3.1 LNP Charge and Cell Culture Medium Composition Affect Transfection Efficiency in Jurkat Cells

**FIGURE 1:**
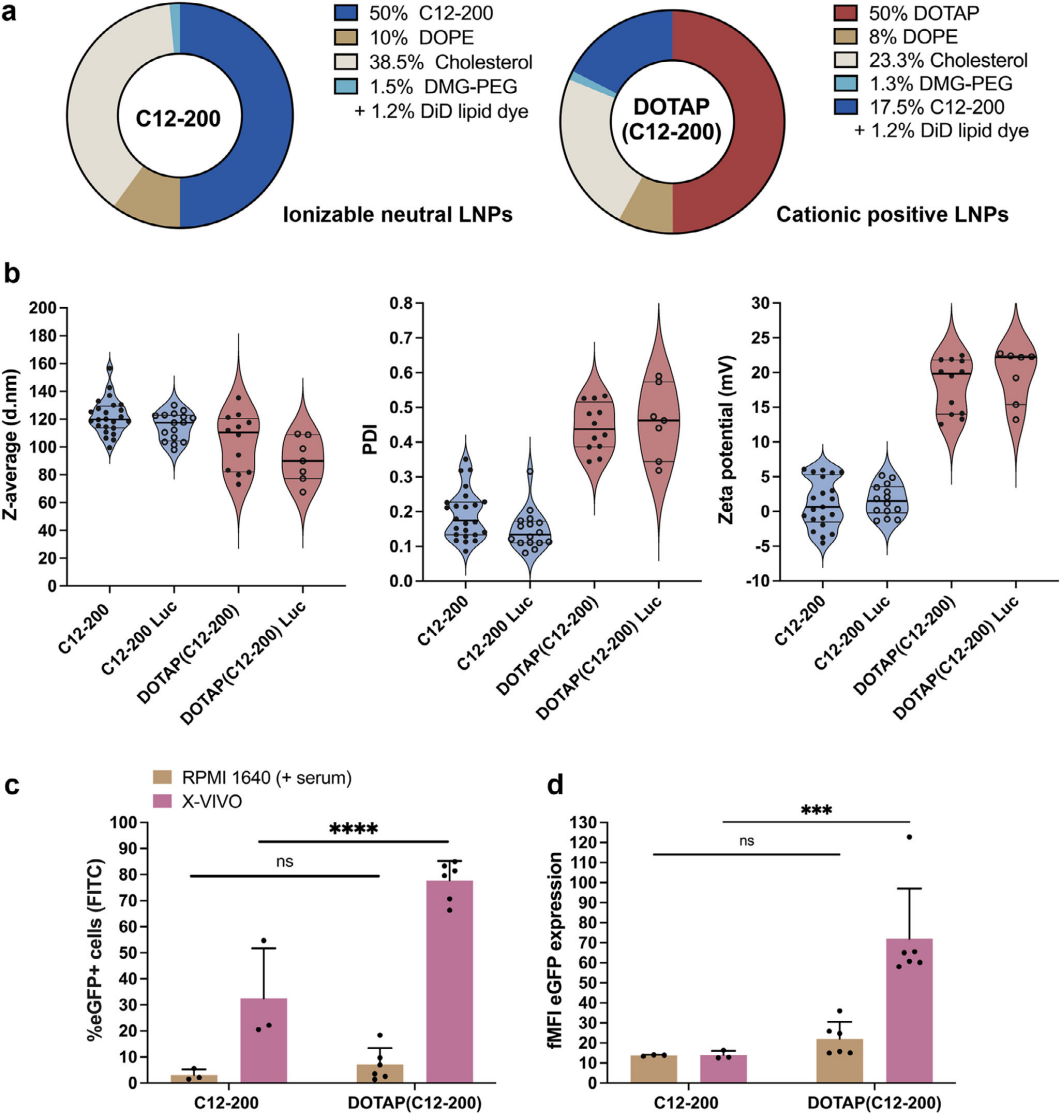
Physicochemical properties and Jurkat cell transfection of C12‐200 and DOTAP(C12‐200) LNPs in different cell culture media. (a) Composition of C12‐200 and DOTAP(C12‐200) lipid nanoparticle (LNP) formulations (mol%). (b) Hydrodynamic diameter (z‐average, d.nm), polydispersity index (PDI), and zeta potential (mV) of C12‐200 (blue) and DOTAP(C12‐200) LNPs (red), encapsulating enhanced green fluorescent protein (eGFP) or firefly luciferase (Luc) mRNA, respectively. Data points represent the mean ± SD of *n* independent formulations with C12‐200 eGFP LNPs (*n* = 24), C12‐200 Luc LNPs (*n* = 16), DOTAP(C12‐200) eGFP mRNA LNPs (*n* = 12), and DOTAP(C12‐200) Luc mRNA LNPs (*n* = 7), respectively. (c) Transfection efficiency and (d) fold mean fluorescence intensity (fMFI) of Jurkat cells after 24 h incubation of 1 ng/µL eGFP mRNA encapsulated in C12‐200 or DOTAP(C12‐200) LNPs, in RPMI 1640 + 10% FBS (Fetal Bovine Serum) and X‐VIVO, respectively. fMFI was calculated as MFI eGFP+ cells/MFI Luc CTR cells. Transfection data are represented as mean ± SD (C12‐200 LNPs: *n* = 3, DOTAP(C12‐200) LNPs: *n* = 6, biological replicates). Statistical analysis was performed using ordinary Two‐way ANOVA with Tukey's multiple comparisons test (ns *p* > 0.05; ^*^ *p* < 0.05; ^**^ *p* < 0.01; ^***^ *p* < 0.001; ^****^ *p* < 0.0001).

Initial screening experiments were performed to evaluate the influence of serum on LNP‐mediated mRNA delivery in T cells. LNPs containing DLin‐MC3‐DMA (MC3), SM‐102, or C12‐200 ionizable lipids were formulated with enhanced green fluorescent protein (eGFP)‐encoding mRNA at state‐of‐the‐art molar ratios (Figure S1). MC3 and SM‐102 were included as clinically validated lipids, while C12‐200 was selected for its previously reported efficiency in pDNA delivery to T cells [14, 27, 46, 47]. Transfection efficiency was evaluated in Jurkat cells, an immortalized human T cell line commonly used in T cell studies, comparing serum‐containing (RPMI 1640 + 10% FBS) and serum‐free (X‐VIVO) medium (Figure S1). Only the C12‐200 LNPs performed significantly better in the serum‐free medium (32.5% eGFP+ cells), compared to the serum‐containing condition (3.1% eGFP+ cells). Next, the impact of a permanently charged cationic lipid on the transfection efficiency of the C12‐200 LNPs was evaluated by adding DOTAP as a fifth component to the quaternary LNP composition (Figure 1a) [36]. Both LNPs were formulated containing mRNA encoding for either eGFP or firefly luciferase (Luc), the latter used as a control to account for any background autofluorescence [48]. An average hydrodynamic diameter of ∼120 nm (*n* = 24) and ∼100 nm (*n* = 12) was measured for C12‐200 LNPs and DOTAP(C12‐200) LNPs, respectively, with the latter typically showing elevated PDI values. As expected, the inclusion of DOTAP shifted the LNP's zeta‐potential from near‐neutral (‐4.5 to 5.9 mV) to a cationic charge at physiological pH (12.6 to 22.5 mV) (Figure 1b). All LNP formulations showed a high mRNA encapsulation efficiency (Table S2). Transfection of Jurkat cells demonstrated that cationic DOTAP(C12‐200) LNPs markedly outperformed their charge‐neutral counterparts in X‐VIVO medium, achieving transfection efficiencies of 77.7 ± 7.5% (mean ± SD, *n* = 6) and 32.5 ± 19.3% (mean ± SD, *n* = 3), respectively. However, when serum was present, both formulations only achieved very low mRNA transfection efficiency (Figure 1c,d).

**FIGURE 2:**
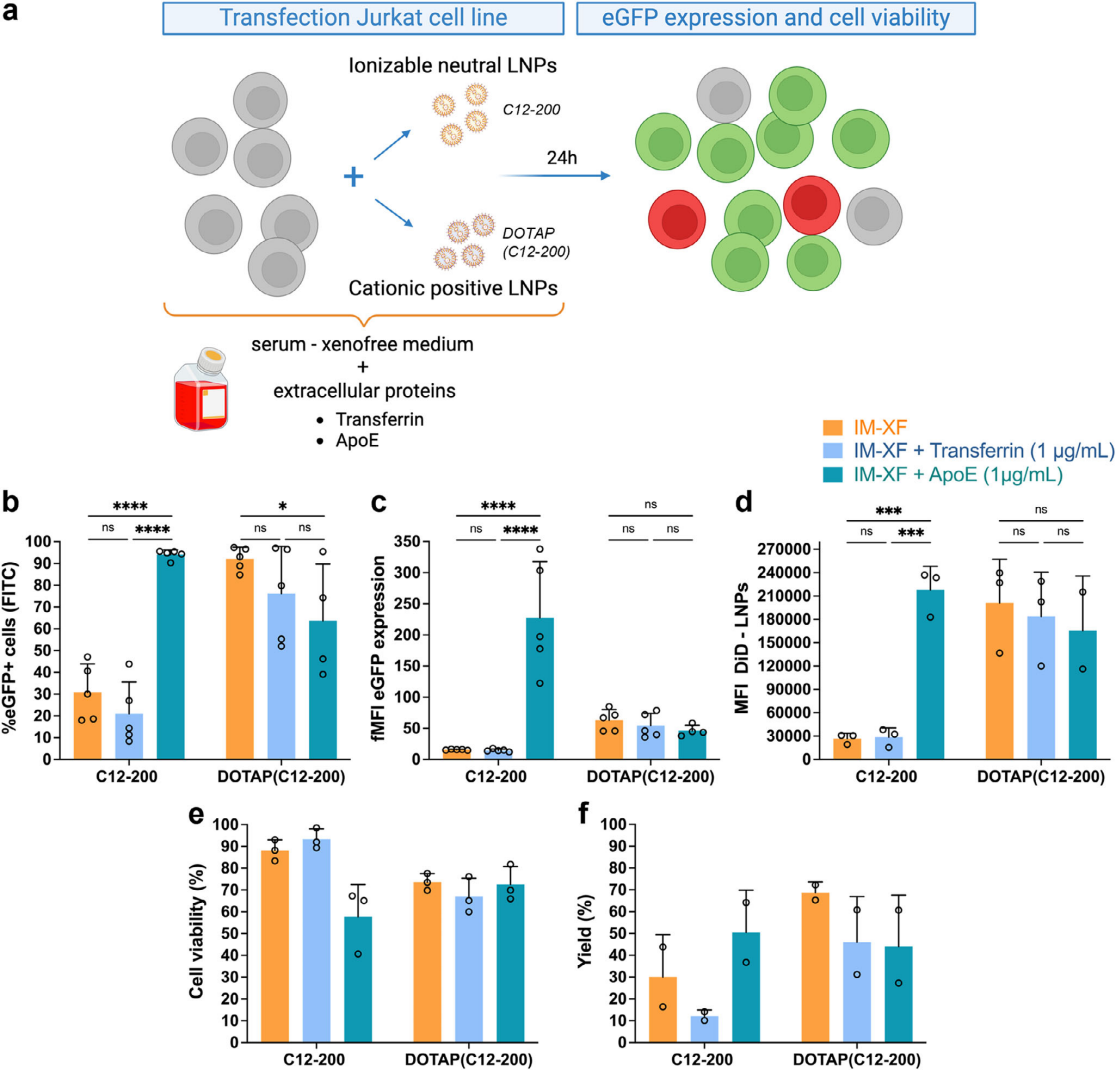
Transfection efficiency of Jurkat cells depends on LNP charge and cell culture medium composition. (a) Schematic overview of experimental procedure. Cells were transfected with either C12‐200 or DOTAP(C12‐200) lipid nanoparticles (LNPs) at a final mRNA concentration of 1 ng/µL per 6 × 10^4^ cells in ImmunoCult‐XF T Cell expansion medium (IM‐XF), IM‐XF + transferrin (1 µg/mL), or IM‐XF + apolipoprotein E (ApoE) (1 µg/mL), respectively. Flow cytometry was used to determine eGFP expression after 24 h incubation. Transfected cells are represented in green, dead cells in red, and non‐transfected cells in grey. (b) Transfection efficiency and (c) fold mean fluorescence intensity (fMFI) of LNPs with or without the presence of additional extracellular proteins (i.e., transferrin and ApoE). fMFI was calculated as MFI eGFP+ cells/MFI Luc CTR cells. Transfection data are presented as mean ± SD (*n* = 5, biological replicates). (d) Mean fluorescence intensity (MFI) of Jurkat cells incubated with DiD‐labeled LNPs. Data are represented as mean ± SD (*n* = 3, biological replicates). (e) Cell viability as determined by CellTiter‐Glo assay after 24 h incubation with LNPs. Data represent mean ± SD (*n* = 3, biological replicates). (f) Transfection yield, calculated by multiplying transfection efficiency (%) and cell viability (%), representing the percentage of viable transfected cells. Data show mean ± SD (*n* = 2, biological replicates). Statistical analysis was performed using ordinary Two‐way ANOVA with Tukey's multiple comparisons test (ns *p* > 0.05; ^*^ *p* < 0.05; ^**^ *p* < 0.01; ^***^ *p* < 0.001; ^****^ *p* < 0.0001). Illustration created in https://BioRender.com.

Having established that the composition of the cell culture medium is an important parameter, possibly through adsorption of proteins to the LNP surface, we next assessed LNP‐mediated Jurkat cell transfection in two different serum‐free T cell culture media (i.e., X‐VIVO and ImmunoCult‐XF T cell expansion medium (IM‐XF)), spiked with either ApoE or transferrin (Figure 2a; Figure S2). ApoE and transferrin can bind to the LDL receptor (LDL‐R) and transferrin receptor (CD71), respectively, both of which are highly expressed on activated T cells and Jurkat cells [49, 50, 51]. In this way, directing the LNPs to these receptors via protein adsorption may lead to enhanced cellular uptake and transfection efficiency. Notably, cationic DOTAP(C12‐200) LNPs transfected Jurkat cells independent of medium composition, while conventional ionizable C12‐200 LNPs specifically required the presence of ApoE in the cell culture medium (Figure 2b; Figure S2). Also, the extent of mRNA expression per cell was significantly higher for C12‐200 LNPs with additional ApoE (Figure 2c). In contrast, additional transferrin had no positive effect on the transfection properties of either LNP. These observations were mirrored in the cell interaction data, representing both LNPs bound to the cell membrane as well as internalized LNPs (Figure 2d). Analogous to transfection efficiency, T cell interaction was improved for C12‐200 LNPs in combination with ApoE protein, while cationic DOTAP(C12‐200) LNPs bound to T cells equally well in all media. In addition, a cell viability above 65%, measured as metabolic activity using the CellTiter‐Glo assay, indicated that the LNPs were relatively well tolerated (Figure 2e). As the quality of the final cell product in cancer immunotherapy is of main importance, the percentage of both viable and transfected cells (i.e., the yield) is a critical parameter. Acceptable yields of 50.5 ± 19.4% and 68.8 ± 4.9% (mean ± SD, *n* = 2) were obtained for respectively C12‐200 LNPs in IM‐XF spiked with ApoE and DOTAP(C12‐200) LNPs in IM‐XF without additional proteins (Figure 2f).

### 3.2 Cationic LNPs Achieve Robust Transfection of Primary T Cells Regardless of Media or Activation State

**FIGURE 3:**
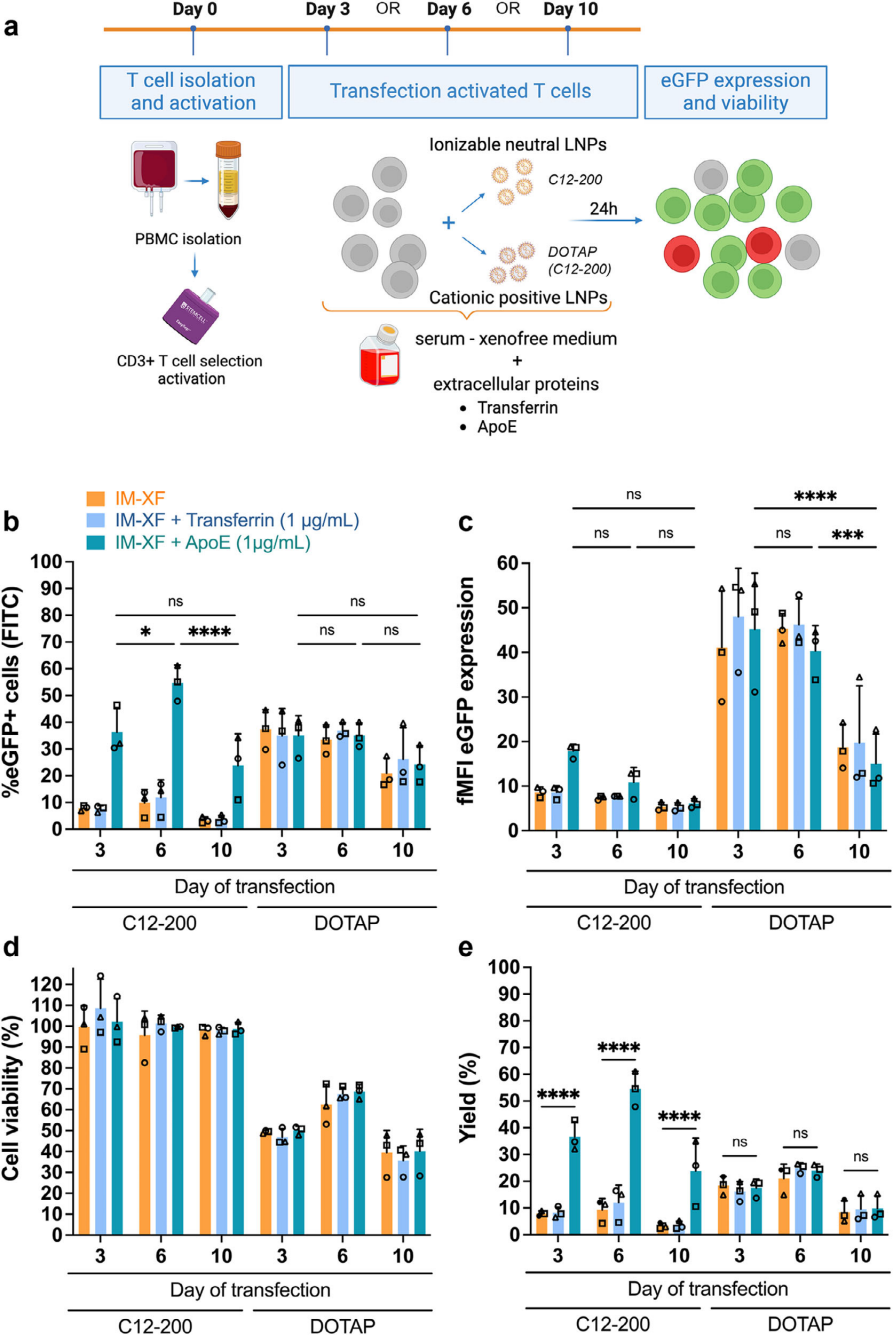
Transfection of primary human T cells depends on LNP charge and available proteins. (a) Schematic representation of experimental workflow. T cells were activated with anti‐CD3/CD28/CD2 antibodies (10 µL/mL) and IL‐2 (10 ng/mL) for up to 10 days. On different time points following activation (i.e., day 3, 6, or 10), T cells were incubated with C12‐200 or DOTAP(C12‐200) lipid nanoparticles (LNPs) in ImmunoCult‐XF T Cell expansion medium (IM‐XF), IM‐XF + transferrin (1 µg/mL), and IM‐XF + apolipoprotein E (ApoE) (1 µg/mL), respectively. A final mRNA concentration of 1 ng/µL for 5 × 10^4^ T cells was used. After 24 h incubation, transfection efficiency and cell viability were determined. (b) Transfection efficiency and (c) fold mean fluorescence intensity (fMFI) determined using flow cytometry. fMFI was calculated as MFI eGFP+ cells/MFI Luc CTR cells. (d) Cell viability was determined using a CellTiter‐Glo assay after 24 h incubation with LNPs. (e) Yield represents the percentage of viable transfected cells and was calculated by multiplying the transfection efficiency and cell viability. Bars show mean ± SD with individual donors represented by different symbols (*n* = 3, biological independent donors). Statistical analysis was performed using ordinary Two‐way ANOVA with Tukey's multiple comparison test (ns *p* > 0.05; ^*^ *p* < 0.05; ^**^ *p* < 0.01; ^***^ *p* < 0.001; ^****^ *p* < 0.0001). Illustration created in https://BioRender.com.

Given that primary human T cells better represent the physiological heterogeneity and activation states found in vivo, C12‐200 and DOTAP(C12‐200) LNPs were next tested in CD3^+^ T cells to validate the findings from Jurkat cells. To this end, T cells were isolated from human buffy coats and subsequently activated using anti‐CD3/CD28/CD2 antibodies in the presence of IL‐2. Next, T cells were transfected on day 3, 6, or 10 post‐activation to compare transfection efficiencies in T cells with different activation state (Figure 3a). The strong increase of CD25 (interleukin 2 receptor alpha chain), CD71 (transferrin receptor) and LDL expression clearly indicated successful T cell activation. However, the receptor density per cell markedly decreased on day 10 post‐activation, suggesting a decline in T cell activation state (Figure S3). Again, DOTAP(C12‐200) LNPs transfected primary T cells equally well independent of the culture medium composition, while the performance of C12‐200 LNPs clearly depended on ApoE, in line with the data obtained on Jurkat cells. Six days after activation, transfection efficiencies of up to 54.7 ± 6.6% and 35.1 ± 4.7% (mean ± SD, *n* = 3) were obtained for respectively C12‐200 and DOTAP(C12‐200) LNPs in IM‐XF with ApoE (Figure 3b). Of note, the extent of mRNA expression was noticeably higher for the cationic DOTAP‐modified LNPs compared to their charge neutral counterparts (Figure 3c). Additionally, other conventional ionizable lipids (i.e., SM‐102 and ALC‐0315) were tested, albeit preliminary results on a single human donor suggest no improvement in transfection efficiency relative to the C12‐200 formulation (Figure S4). Replacement of cholesterol by β‐sitosterol, a naturally occurring cholesterol analogue, was previously reported to improve transfection efficiency of NK cells by enhancing endosomal escape [52, 53]. However, our data suggest that inclusion of β‐sitosterol in C12‐200 LNPs does not further promote mRNA expression in primary T cells (Figure S5).

The optimal time point for LNP transfection of T cells after activation remains poorly defined, yet it may critically influence uptake and expression efficiency. For C12‐200 LNPs in IM‐XF + ApoE, T cells transfected on day 6 post‐activation showed the highest transfection efficiency (54.7% ± 6.6) compared to T cells transfected on day 3 (36.3 ± 8.8%) or day 10 (23.9 ± 11.8%) post‐activation (mean ± SD, *n* = 3) (Figure 3b). However, eGFP expression levels, measured as fold mean fluorescence intensity (fMFI), were slightly higher on day 3, although not significant (Figure 3c). In contrast, DOTAP(C12‐200) LNPs were less affected by activation time, showing no significant differences in transfection efficiency at different time points (Figure 3b), albeit fMFI values were clearly lower on day 10 (Figure 3c). Opposed to C12‐200 LNPs, DOTAP(C12‐200) LNPs also appeared to be partly cytotoxic, with remaining fractions of viable cells, as determined with CellTiter‐Glo, ranging from 35.7% to 68.7%, depending on the day of transfection (Figure 3d). Hence, judging from the final yield of viable transfected cells, the combination of C12‐200 LNPs in IM‐XF + ApoE was identified as the most optimal transfection condition on day 3 and 6 (36.6 ± 5.7% [day 3]; 54.5 ± 6.6% [day 6]) (mean ± SD, *n* = 3). Similar to transfection efficiency, no significant differences regarding yield were observed for DOTAP(C12‐200) LNPs used in distinct transfection media (Figure 3e).

**FIGURE 4:**
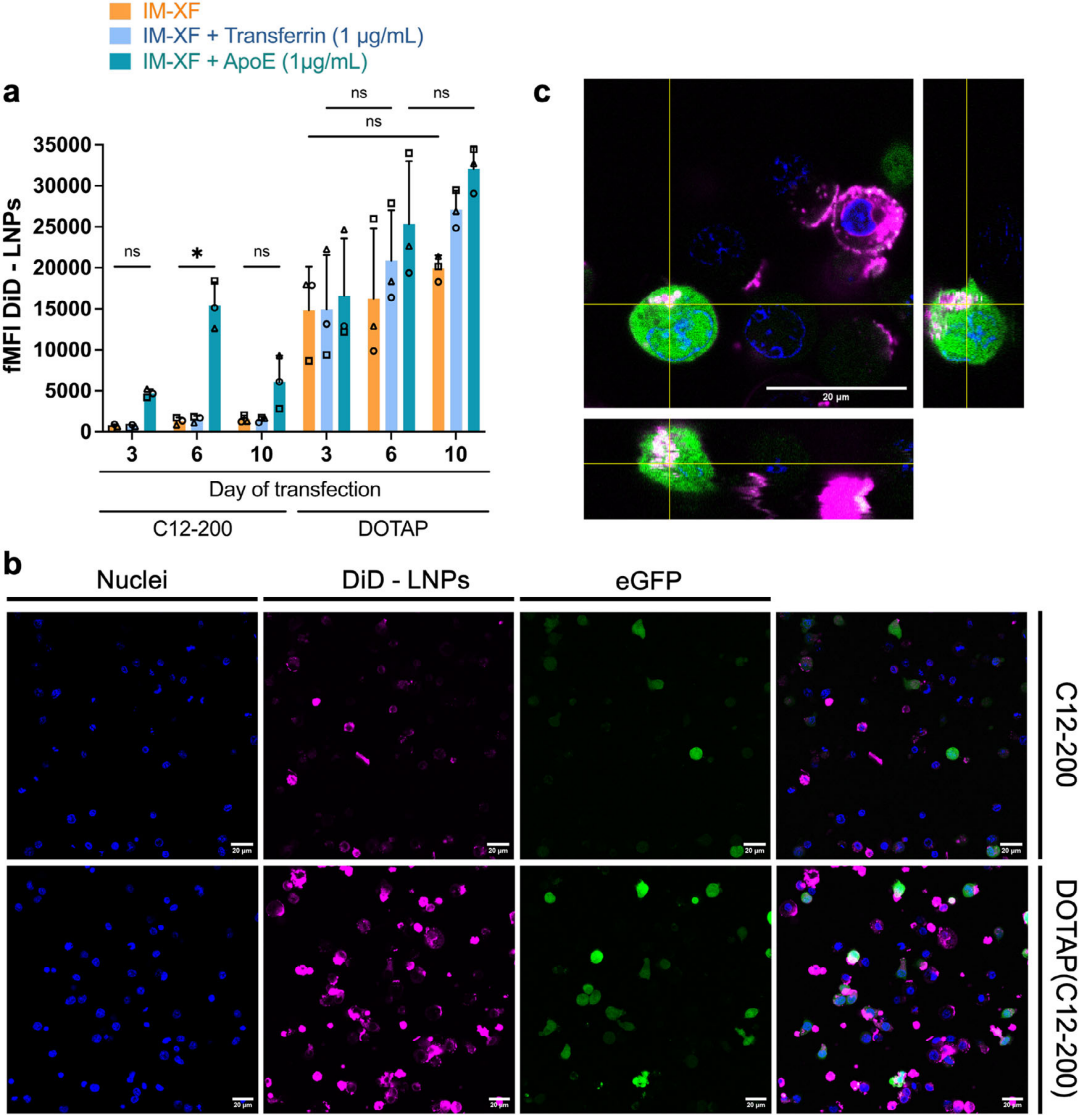
LNP interaction with primary human T cells depends on LNP composition and cell culture proteins. (a) T cells transfected in ImmunoCult‐XF T Cell expansion medium (IM‐XF), IM‐XF + apolipoprotein E (ApoE) (1 µg/mL), or IM‐XF + transferrin (1 µg/mL) on day 3, 6, or 10 post‐activation using C12‐200 and DOTAP(C12‐200) lipid nanoparticles (LNPs) containing 1.2 mol% DiD‐lipid dye at a dose of 1 ng/µL for 5 × 10^4^ T cells. After 24 h incubation, fold mean fluorescence intensity (fMFI) of DiD‐LNPs was determined with flow cytometry for the evaluation of LNP‐T cell interaction. fMFI was calculated as MFI DiD+ cells/MFI Luc CTR cells. Data represent mean ± SD with individual donors represented by different symbols (*n* = 3, biological independent donors). Statistical analysis was performed using ordinary Two‐way ANOVA with Tukey's multiple comparison test (ns *p* > 0.05; ^*^ *p* < 0.05; ^**^ *p* < 0.01; ^***^ *p* < 0.001; ^****^ *p* < 0.0001). (b) Confocal microscopy images of activated primary T cells transfected with DiD‐labeled LNPs (C12‐200 and DOTAP(C12‐200)) containing eGFP mRNA. Hoechst nuclear staining is presented in blue, DiD‐LNPs in magenta, and eGFP expression in green. T cells were transfected on day 3 after initial activation with 1 ng/µL mRNA for both C12‐200 and DOTAP(C12‐200) LNPs in IM‐XF medium. (c) Orthogonal (XY‐XZ) representation of a confocal Z‐stack of activated primary human T cells transfected with DiD‐labeled DOTAP(C12‐200) LNPs in IM‐XF medium at a concentration of 1 ng/µL eGFP mRNA. Scale bar represents 20 µm.

Higher T cell binding and internalization was also observed for C12‐200 LNPs in the presence of ApoE (Figure 4a). Moreover, LNP‐T cell interaction was highest for T cells transfected on day 6 post‐activation. These results mirrored the percentage of eGFP+ cells, again correlating cell interaction with mRNA expression (Figures 3b, 4a). In contrast, DOTAP(C12‐200) LNP binding was generally higher compared to C12‐200 LNPs, although no significant difference in LNP binding was observed between different media and days of transfection (Figure 4a). Nevertheless, high variation in the results was noted, most likely induced by biological donor variability. Confocal imaging supported these conclusions, with representative images shown in Figure 4b. Of note, DOTAP(C12‐200) LNPs seemed to be present at the cell plasma membrane, possibly via unspecific electrostatic interaction with the negatively charged cell surface, as well as inside the cells (Figure 4c). Microscopy images further indicated that not all T cells showing a high degree of interaction with LNPs also had high mRNA expression.

### 3.3 Dose Increase of Non‐Toxic C12‐200 LNPs Improves T Cell Transfection Yield

**FIGURE 5:**
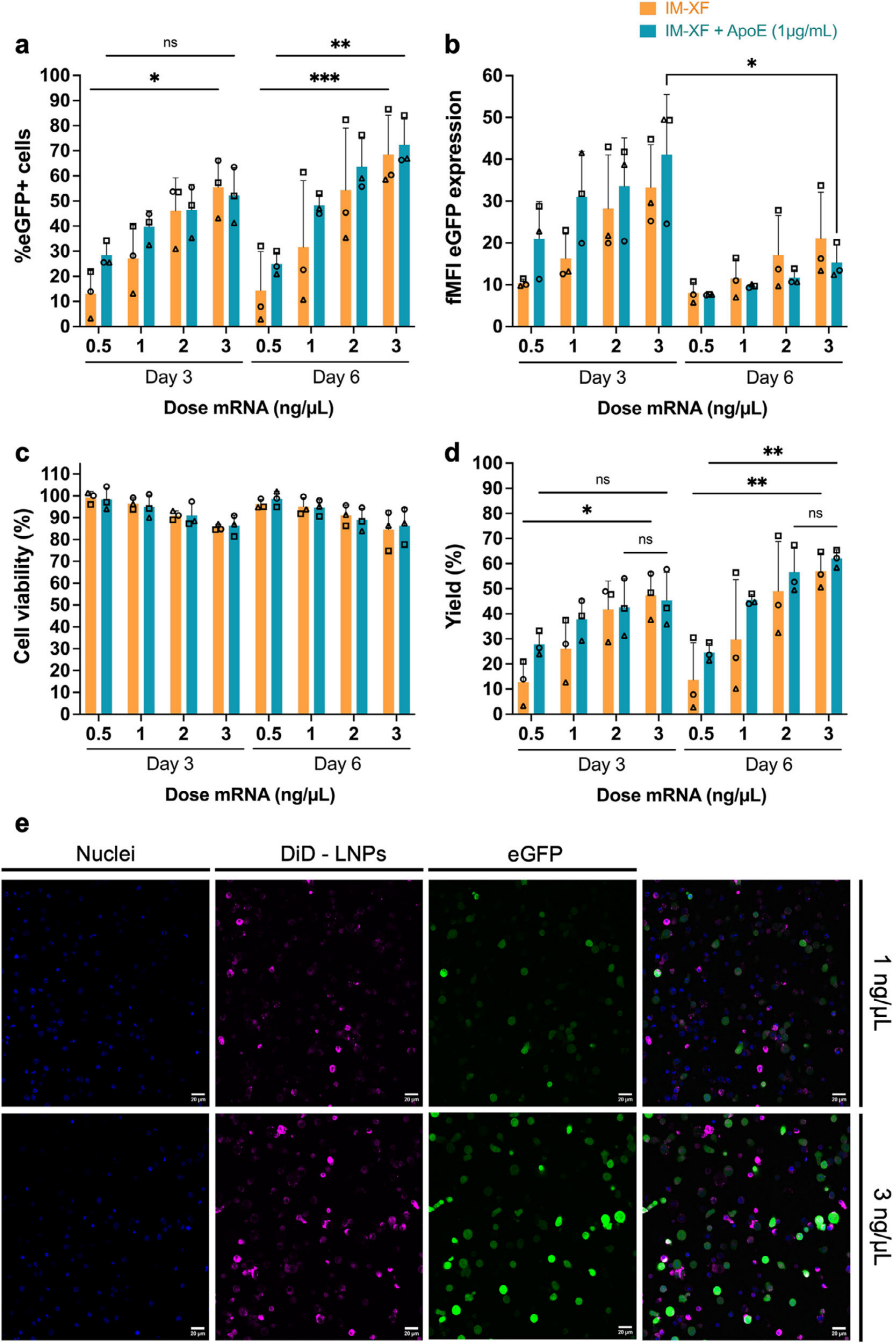
mRNA dose titration for C12‐200 LNPs increases transfection efficiency without inducing high toxicity. (a) Evaluation of transfection efficiency and (b) fold mean fluorescence intensity (fMFI) of eGFP expression using flow cytometry. T cells were transfected on day 3 or day 6 after activation (isolation and activation: day 0) with an increasing dose of C12‐200 lipid nanoparticles (LNPs) (i.e., 0.5, 1, 2, 3 ng/µL mRNA per 5 × 10^4^ T cells in 100 µL) in ImmunoCult‐XF T Cell expansion medium (IM‐XF) and IM‐XF + apolipoprotein E (ApoE), respectively. Flow cytometry was performed after 24 h of incubation with the LNPs. fMFI was calculated as MFI eGFP+ cells/MFI Luc CTR cells. (c) Cell viability was assessed using a CellTiter‐Glo assay following 24 h incubation with the LNPs. (d) Cell yield, indicating the percentage of viable transfected cells, as calculated by multiplying the percentage of eGFP‐positive cells with the cell viability. (e) Representative confocal images of activated CD3^+^ T cells 24 h after transfection with eGFP mRNA‐loaded C12‐200 LNPs. Transfection was performed on day 3, and mRNA doses of 1 ng/µL or 3 ng/µL were used in IM‐XF medium. Nuclei (Hoechst), C12‐200 LNPs (DiD dye), and eGFP expression are colored in blue, magenta, and green, respectively. Scale bars correspond to 20 µm (zoomed images) or 50 µm (overview image). Data represent mean ± SD (*n* = 3 biological independent donors, each represented by a different symbol). Statistical analysis was performed using ordinary Two‐way ANOVA with Tukey's multiple comparison test (ns *p* > 0.05; ^*^ *p* < 0.05; ^**^ *p* < 0.01; ^***^ *p* < 0.001; ^****^ *p* < 0.0001).

As previous data showed that C12‐200 LNPs did not affect cell viability (Figure 3d), an mRNA dose titration was performed to further optimize T cell transfection yield. T cells at day 3 and 6 post‐activation were treated with mRNA LNP doses ranging from 0.5 up to 3 ng/µL. Despite high inter‐donor variability, an increasing trend in T cell interaction was observed with mounting mRNA LNP doses (Figure S6). A dose‐dependent increase in both percentage of transfected cells and eGFP expression per cell was likewise noted, both in IM‐XF and IM‐XF + ApoE, reaching 55.5 ± 11.6% eGFP+ cells at the highest dose tested with an fMFI of 33.2 ± 10.3 [IM‐XF] (Figure 5a,b). Similar to previous data, transfection on day 6 typically provided a higher fraction of eGFP+ cells, but with a lower eGFP signal per cell as compared to day 3. Interestingly, upon increased mRNA doses, the transfection advantage of adding ApoE to the cell culture medium decreased. Furthermore, even at the highest mRNA LNP dose tested, only limited cytotoxicity was observed (∼85% viable cells) (Figure 5c), leading to a final cell yield of ∼60% (Figure 5d). Confocal microscopy confirmed the increase in T cell interaction and eGFP expression at higher mRNA doses (Figure 5e). Taken together, these findings identified 3 ng/µL as the optimal transfection condition, balancing transfection efficiency and cell viability.

### 3.4 Kinetics of mRNA Expression Influenced by the Presence of ApoE Protein

**FIGURE 6:**
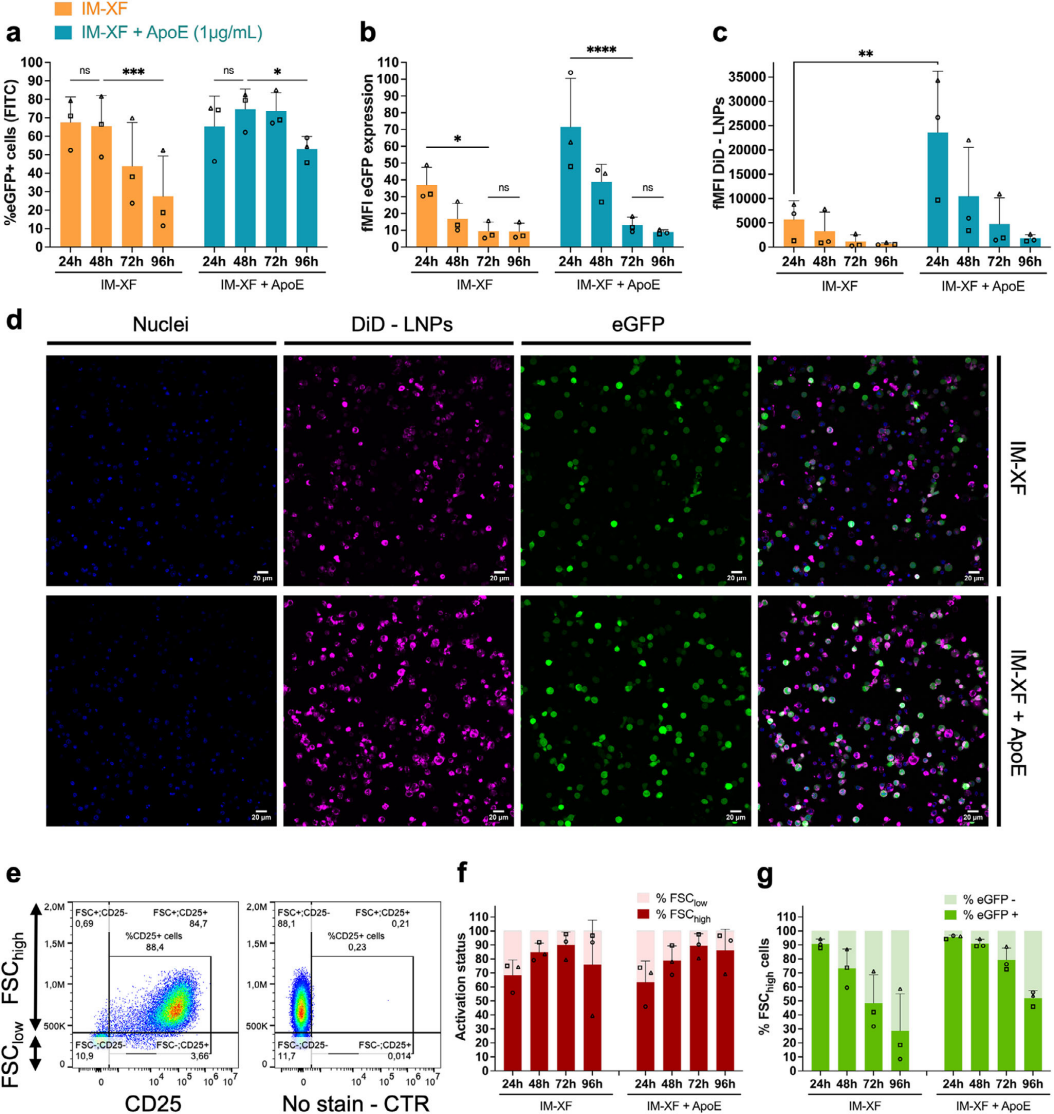
Kinetics of mRNA‐encoded eGFP expression in C12‐200 LNP‐transfected primary human T cells. eGFP protein expression in CD3^+^ T cells was determined at different time points post‐transfection. Transfections were performed with DiD‐labeled C12‐200 lipid nanoparticles (LNPs) at a dose of 3 ng/µL eGFP mRNA in ImmunoCult‐XF T Cell expansion medium (IM‐XF) and IM‐XF + apolipoprotein E (ApoE) (1 µg/mL), respectively. (a) Transfection efficiency and (b) fold mean fluorescence intensity (fMFI) of eGFP expression evaluated on set time points (i.e., 24, 48, 72, 96 h) after initial transfection. (c) LNP‐T cell interaction was determined with flow cytometry and represented by the fMFI of DiD fluorescence. (d) Confocal microscopy images representing activated CD3^+^ T cells 24 h after transfection with DiD‐labeled C12‐200 LNPs at a dose of 3 ng/µL eGFP mRNA. Signals from nuclei, DiD‐LNPs, and eGFP expression are color‐coded in blue, magenta, and green, respectively. Scalebar represents 20 µm. (e) Scatterplots of CD3^+^ T cells labeled with CD25‐APC antibody on day 6 after activation. Gating was set to distinguish between FSC_high_ and FSC_low_ T cell populations. (f) Activation status of T cells is evaluated by the difference in forward scatter (FSC) determined during flow cytometric analysis. (g) Transfection efficiency represented as the % eGFP‐expressing cells in the FSC_high_ population. Data represent three biological independent donors (*n* = 3). Statistical analysis was performed using a mixed‐effects model to account for repeated measurements (matched values), with time after transfection and transfection medium as fixed effects and donor variability as a random effect. Post‐hoc comparisons between time points and between media were performed using Tukey's multiple comparisons (a,b) or Šidák's multiple comparisons test (c). Significant differences are indicated by asterisks (ns *p* > 0.05; ^*^ *p* < 0.05; ^**^ *p* < 0.01; ^***^ *p* < 0.001; ^****^ *p* < 0.0001). Error bars represent mean ± SD, *n* = 3 biologically independent donors.

Next, it was evaluated how long mRNA expression lasts in T cells upon delivery with C12‐200 LNPs. Therefore, activated T cells were transfected on day 3 post‐activation with C12‐200 LNPs in IM‐XF medium with or without ApoE (1 µg/mL) at an mRNA dose of 3 ng/µL. After 24 h incubation, LNPs were removed, and cells were re‐stimulated in IM‐XF with anti‐CD3/CD28/CD2 antibodies. A significant decrease in the percentage of eGFP+ T cells was observed from 48 to 96 h after transfection with C12‐200 LNPs, albeit transfected cells remained detectable up until 96 h (Figure 6a). Additionally, fMFI values indicated that the intensity of eGFP expression decreased significantly after 72 h for T cells transfected in both IM‐XF and IM‐XF + ApoE (Figure 6b). Nevertheless, in line with previous observations, ApoE clearly improved the extent of eGFP expression, enabling to maintain higher mRNA expression at later time points post‐transfection (Figure 6a,b; Figure S7). Moreover, the presence of ApoE in the cell culture medium promoted T cell binding, as confirmed both quantitatively with flow cytometry as well as qualitatively on confocal microscopy images (Figure 6c,d).

We then aimed to examine the role of T cell activation on transfection efficiency. Previous experiments revealed that the activation marker CD25 is expressed only in cells with a high forward scatter (FSC) (Figure 6e; Figure S8). Therefore, the transfection data were re‐analyzed to distinguish between FSC_high_ and FSC_low_ cells as a proxy for T cell activation status (Figure 6f). Notably, eGFP expression was observed only in the FSC_high_ cell population, indicating that only activated T cells are susceptible to C12‐200 LNP transfection (Figure S7). When solely the FSC_high_ population was considered, most T cells were clearly transfected (90.8 ± 3.3% in IM‐XF and 95.7 ± 1.3% in IM‐XF + ApoE (mean ± SD, *n* = 3) (Figure 6g). Altogether, our findings indicate that T cell activation is essential for robust C12‐200 LNP transfection and that supplementation of the cell culture medium with ApoE protein further stimulates both C12‐200 LNP uptake and transfection, thereby extending mRNA expression.

### 3.5 Ionizable and Cationic LNPs Can Efficiently Deliver nanoCAR mRNA in Activated T Cells

Having determined the optimal conditions for LNP transfections of CD3^+^ T cells using an eGFP mRNA model, the neutral C12‐200 and cationic DOTAP(C12‐200) LNPs were used to transfect T cells with anti‐CD20 nanoCAR mRNA. Patients with relapsed or refractory B‐cell non‐Hodgkin lymphomas exhibit elevated levels of the CD20 antigen on their B cells, making CD20 CAR‐T cells a plausible treatment strategy [54, 55]. Moreover, both preclinical and clinical studies have shown promising results for the use of CD20 CAR‐T cells in treating autoimmune diseases, such as multiple sclerosis (MS) and systemic lupus erythematosus (SLE), by depleting autoreactive B cells [56, 57]. NanoCAR‐T cells are engineered with CAR constructs that use nanobodies as the antigen‐binding domain instead of conventional single‐chain variable fragments (scFvs) [44]. Nanobodies are derived from the V_H_ domain of heavy‐chain only antibodies (HcAbs) found in Camelidae, which lack the constant heavy 1 (CH1) domain and therefore do not have a light chain, resulting in a single antigen‐binding domain and not two [58]. Their small size, low immunogenicity, and strict monomeric behavior are making them an attractive alternative to scFv‐based CARs, with the added potential to design tandem CARs to address immune escape and relapse after CAR‐T cell infusion [44, 58].

**FIGURE 7:**
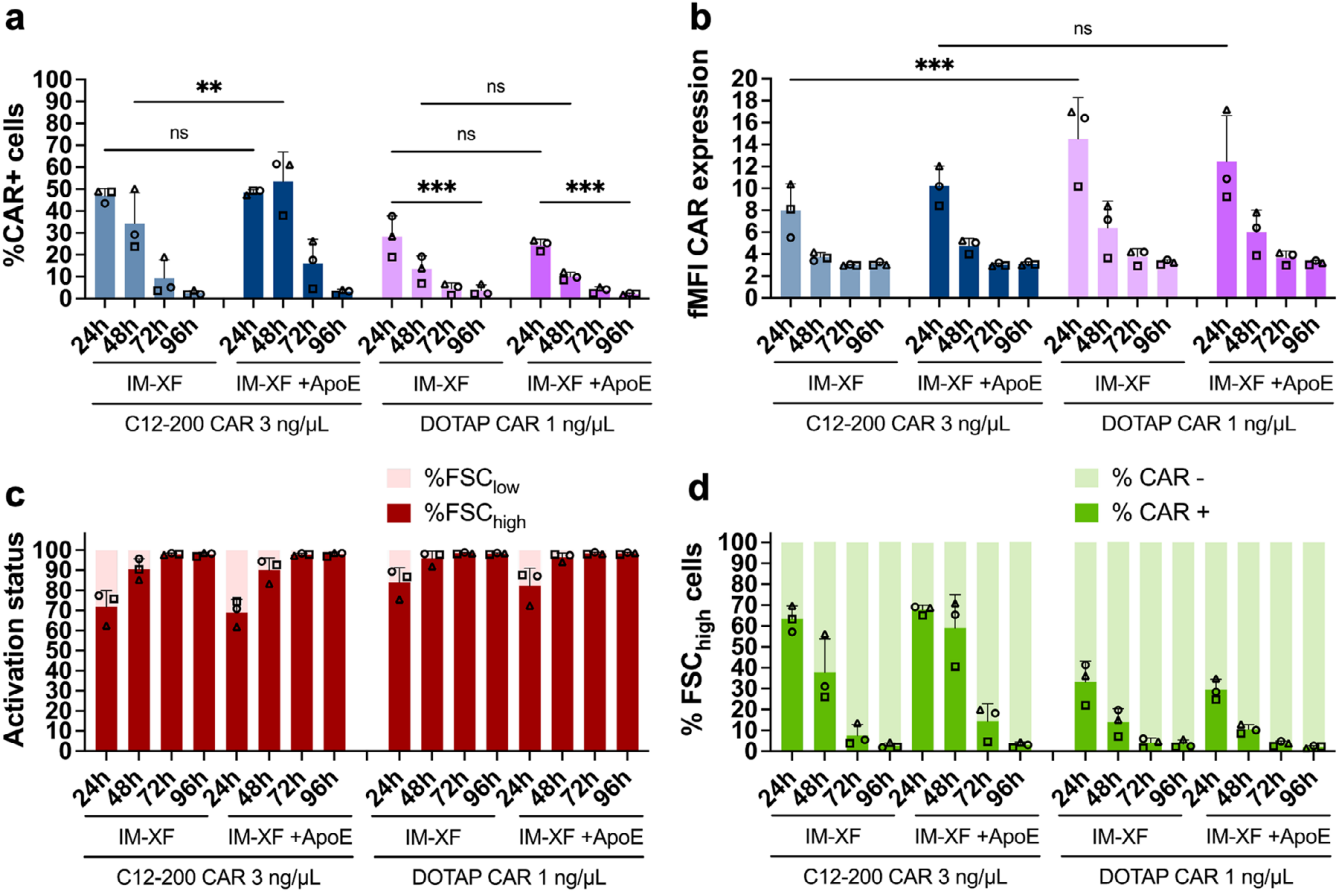
LNP‐mediated delivery of anti‐CD20 nanoCAR mRNA allows for efficient nanoCAR expression in activated T cells. Ionizable C12‐200 and cationic DOTAP(C12‐200) lipid nanoparticles (LNPs) were used for the delivery of anti‐CD20 nanoCAR mRNA in activated T cells on day 3 after initial stimulation. Experiments were performed in ImmunoCult‐XF T Cell expansion medium (IM‐XF) or IM‐XF + apolipoprotein E (ApoE) (1 µg/mL) using a dose of 3 and 1 ng/µL mRNA for C12‐200 and DOTAP(C12‐200) LNPs, respectively. Flow cytometry was performed to determine (a) % nanoCAR+ cells in the total population, (b) fold median fluorescence intensity (fMFI) calculated as (MFI CAR+ cells—MFI Mock live cells)/(MFI CTR live cells). (c) and activation status. (d) % nanoCAR+ forward scatter high (FSC_high_) cells represented as the ratio of % nanoCAR+ cells in the FSC_high_ population divided by % FSC_high_ cells. A mixed‐effects model was used for statistical analysis to account for repeated measures (matched values). Time after transfection in the specified medium and LNP treatment are described as fixed effects and donor variability as a random effect. Post‐hoc comparisons between time points or between LNP treatments were performed using Tukey's (a) or Šidák's multiple comparisons test (b). Significant differences are indicated by asterisks (ns *p* > 0.05; ^*^ *p* < 0.05; ^**^ *p* < 0.01; ^***^ *p* < 0.001; ****p < 0.0001). Error bars represent mean ± SD, *n* = 3 biologically independent donors.

Here, evaluation of the percentage of nanoCAR+ cells revealed no significant differences between IM‐XF and IM‐XF + ApoE for the ionizable C12‐200 LNPs after 24 h. However, spiking the culture medium with ApoE allowed for a prolonged nanoCAR expression until 48 h post‐transfection, providing up to 34.3 ± 13.9% and 53.5 ± 13.5% nanoCAR+ T cells in IM‐XF and IM‐XF + ApoE, respectively (mean ± SD, *n* = 3) (Figure 7a). On the other hand, no significant differences could be detected between either media for cationic DOTAP(C12‐200) LNPs, confirming previous findings (Figure 7a). Despite DOTAP(C12‐200) LNPs providing a lower percentage of nanoCAR+ cells compared to C12‐200 LNPs (i.e., 28.4 ± 9.3% versus 47.1 ± 3.1%, respectively [24 h, IM‐XF, mean ± SD, *n* = 3]), DOTAP(C12‐200) LNPs at 1 ng/µL still generated a higher nanoCAR expression per transfected cell than C12‐200 LNPs at 3 ng/µL (Figure 7b). Due to a clear reduction in cell viability and yield observed earlier, the dose of DOTAP(C12‐200) LNPs was not further increased to 3 ng/µL (Figure S9a,b). Nevertheless, despite a lower cell viability 24 h after DOTAP(C12‐200) LNP treatment, the T cells exhibited similar proliferation kinetics to C12‐200 LNP‐treated and untreated activated T cells (Figure S9c).

Next, we again evaluated the impact of T cell activation on LNP‐mediated nanoCAR expression, by gating on FSC_high_ and FSC_low_ cell populations. All transfection conditions achieved up to 98% FSC_high_ T cells after 96 h in culture (Figure 7c). Evaluation of nanoCAR expression in the FSC_high_ subpopulation showed an increase of nanoCAR+ cells from 47.1 ± 3.1% to 63.4 ± 6.3% for C12‐200 LNPs after 24 h incubation in IM‐XF (mean ± SD, *n* = 3). Contrarily, no significant increase was observed for DOTAP(C12‐200) LNP‐treated T cells (Figure 7a,d). The latter can likely be explained by the higher toxicity of DOTAP(C12‐200) LNPs specifically toward unstimulated FSC_low_ T cells (Figure S10b). Overall, our results showed that both C12‐200 and DOTAP(C12‐200) LNPs under optimized conditions are suitable to deliver and express anti‐CD20 nanoCAR in activated T cells.

### 3.6 Transiently Engineered nanoCAR‐T Cells Exhibit Tumor Cell Killing

**FIGURE 8:**
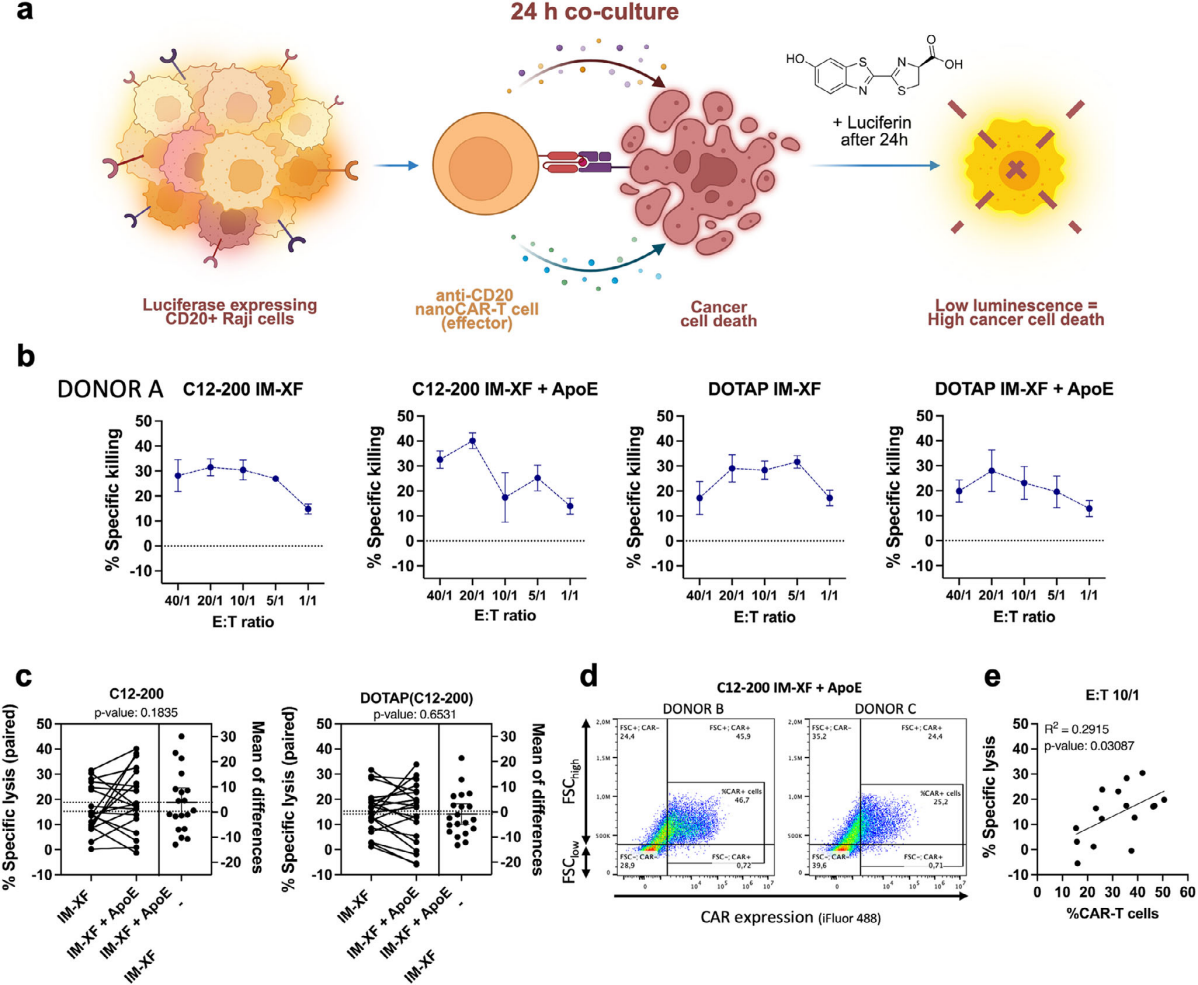
Functional cytolytic capacities for transiently engineered CD20‐specific nanoCAR‐T cells. (a) Overview of experimental workflow for in vitro Raji cell co‐culture assay. (b) Specific cell lysis (%) of Raji target cells expressing CD20 after 24 h incubation with T cells expressing CD20 nanoCAR in different effector‐to‐target (E:T) ratios. eGFP mRNA lipid nanoparticles (LNPs) were used as a control, and a correction for background lysis was performed. Reported data represent the mean of technical triplicates ± standard error of the mean (SEM). Results are representative of two independent experiments, performed with four different donors. (c) Estimation plot showing specific lysis after engineering of nanoCAR‐T cells in ImmunoCult‐XF T Cell expansion medium (IM‐XF) or IM‐XF + apolipoprotein E (ApoE) (1 µg/mL) for C12‐200 and DOTAP(C12‐200) LNPs, respectively. Each dot represents an independent donor at a specified E:T ratio (i.e., 40/1, 20/1, 10/1 or 5/1), with lines connecting paired measurements. The right panel displays the mean difference with a 95% confidence interval (CI). Statistical analysis was performed using a two‐tailed, paired *t*‐test. (d) Scatterplots of nanoCAR+ T cells labeled with iFluor488 VHH nanobody 24 h after transfection with C12‐200 LNPs in IM‐XF + ApoE (1 µg/mL) for two independent biological donors (Donor B vs. C). Gating was set to distinguish between forward scatter high (FSC_high_) and FSC_low_ T cell populations. (e) Correlation between % anti‐CD20 nanoCAR‐T cells and specific cytolytic activity (%) after 24 h co‐culture with CD20^+^ Raji cells. A significant positive correlation was observed for E:T ratio 10/1 (*r = 0.54, p = 0.03, R^2^ = 0.29*) when combining all treatment conditions. The solid line represents the best‐fit linear regression. Data represents *n* = 4 independent biological donors. Figure created in https://BioRender.com.

The cytolytic activity of the transiently engineered nanoCAR‐T cells was evaluated in an in vitro co‐culture assay with CD20^+^ Raji cells stably expressing firefly luciferase. T cells transfected with either C12‐200 or DOTAP(C12‐200) LNPs encapsulating anti‐CD20 nanoCAR mRNA were harvested after 24 h incubation in IM‐XF or IM‐XF + ApoE medium. Subsequently, the transfected T cell population was counted and co‐cultured with Raji target cells for 24 h at effector‐to‐target (E:T) ratios of 40:1, 20:1, 10:1, 5:1, and 1:1. Here, effector cells included both nanoCAR+ T cells as well as untransfected T cells. Tumor cell killing was determined by the decrease in luminescence upon cancer cell death as compared to the luminescent signal from unchallenged Raji cells (Figure 8a) [59]. T cells transfected with LNPs encapsulating eGFP mRNA acted as mock controls to assess unspecific lysis due to the highly active T cell state at the time of the assay (Figure S11). Therefore, specific lysis was calculated as the cell lysis of nanoCAR‐transfected T cells subtracted by the percentage cell lysis induced by mock‐transfected T cells. Specific cell killing was observed for C12‐200 and DOTAP(C12‐200) LNP‐transfected T cells in both transfection media (i.e., IM‐XF and IM‐XF + ApoE), with up to 40% nanoCAR‐specific cell lysis (C12‐200 LNP, IM‐XF + ApoE, E:T 20/1) (Figure 8b; Figure S12). No significant improvement of specific tumor cell lysis was obtained when comparing nanoCAR‐T cells transfected in medium with or without ApoE (*p* = 0.1835 for C12‐200 LNPs and *p* = 0.6531 for DOTAP(C12‐200) LNPs) (Figure 8c). This result was expected for DOTAP(C12‐200) LNPs as adding ApoE to the culture medium did not improve nanoCAR expression (Figure 7a; Figure S13). Although T cells engineered with C12‐200 LNPs in IM‐XF + ApoE did show an extended nanoCAR expression up to 48 h (Figure 7a), the relatively modest increase in nanoCAR expression obtained by ApoE (Figure S13) may be insufficient to enhance tumor cell killing. Nevertheless, enhancement of transfection efficiency by ApoE appeared to be donor‐dependent, as reflected by the variable percentage of nanoCAR‐T cells in IM‐XF vs. IM‐XF + ApoE at the time of the killing assay (Figure 8d; Figure S13). Of note, a more efficient transfection led to a higher cytolytic activity for a 10/1 E:T ratio, with a clear positive correlation between the percentage of transfected nanoCAR‐T cells and specific cell lysis (*p* = 0.031) (Figure 8e; Figure S14). However, for higher E:T ratios (e.g., 40/1), no such correlation was found (*p* = 0.75; Figure S14), possibly due to more non‐specific killing by mock‐transfected activated T cells. Likewise, the correlation for the lowest E:T ratio of 1/1 was non‐significant (*p* = 0.60), as low levels of nanoCAR‐T killing are likely obscured by high donor variability (Figures S11, S12, S14).

## 4 Discussion

It is known that proteins can electrostatically interact with specifically charged lipids depending on their isoelectric point (pI), which can be exploited for receptor‐mediated cell targeting [39]. For example, the cationic DOTAP lipid is believed to promote the adsorption of proteins with a pI lower than the physiological pH of 7.4 upon intravenous administration [39, 60]. In contrast, our ex vivo transfection results have shown that cationic DOTAP(C12‐200) LNPs transfect T cells independent from the presence of proteins with acidic pI, i.e., both ApoE (pI ∼5.6) and transferrin (pI ∼6.7). Nevertheless, it was verified that both CD71 (transferrin receptor) and LDL‐R (ApoE receptor) are upregulated following T cell activation, in line with the literature [61, 62]. Moreover, the transferrin receptor is involved in clathrin‐mediated endocytosis (CME), and a previous study has shown improved siRNA delivery using transferrin‐PEI polyplexes in activated T cells [63, 64, 65]. Hence, alternative transfection mechanisms for DOTAP‐modified LNPs might be involved, including charge‐based interaction with the anionic cell membrane or cellular entry through macropinocytosis, both of which do not require specific receptor‐mediated targeting [64, 66, 67]. The involvement of the cationic charge is further supported by the inability of DOTAP(C12‐200) LNPs to transfect Jurkat cells in the presence of serum, likely due to the positive surface charge being shielded by the random adsorption of serum proteins. Of note, it was also shown earlier that the addition of ApoE does not improve cellular uptake of cationic LNPs and that cationic DOTAP‐modified LNPs predominantly acquire other serum proteins with acidic pI (i.e., vitronectin) on their surface when injected in the blood circulation [38, 39, 40]. Both confocal microscopy and flow cytometry confirmed a more intense interaction between DOTAP(C12‐200) LNPs and T cells compared to C12‐200 LNPs, which could explain both the markedly higher mRNA expression per cell as well as reduced cell viability observed for the cationic LNPs. Previous research has indicated that the concentration and type of cationic lipid used in lipid‐based formulations may have a profound impact on cytotoxicity [68, 69, 70].

Opposed to cationic LNPs, ApoE is known to interact with the surface of charge‐neutral ionizable LNPs (e.g., DLin‐KC2‐DMA LNPs) and promote transfection efficiency via LDL‐R mediated endocytosis and improved cytosolic delivery [40, 46, 71]. Literature also shows that the presence of DOPE helper lipids in the C12‐200 LNPs contributes to a strong ApoE‐LNP interaction [72]. Here, we likewise demonstrated that ionizable C12‐200 LNPs generally require the presence of ApoE for improved T cell transfection, which may be driven by enhanced LDL‐R expression upon T cell activation [61]. Nevertheless, additional experiments would be required to validate LDL‐R involvement. Consequently, the presence of an activation step in the manufacturing workflow, as well as the time post‐activation are of crucial importance to obtain an optimal transfection efficiency. In contrast, the timing of transfection appears less important for cationic DOTAP(C12‐200) LNPs, again supporting a passive charge‐based rather than an active receptor‐mediated transfection mechanism. The ability to transfect less activated T cell phenotypes could hold a therapeutic advantage. Studies have shown that a shorter ex vivo culture period is associated with improved CAR T cell functionality by providing less differentiated T cells and reducing the risk of T cell exhaustion [41, 73, 74, 75]. Hence, such an accelerated manufacturing workflow can substantially reduce the treatment cost and vein‐to‐vein time, thus increasing treatment accessibility and avoiding disease progression in critically ill patients prior to receiving the CAR‐T cell product [43, 75, 76]. Of note, activation of the T cells does remain necessary, as our results suggest no transfection of non‐proliferating T cells.

Anti‐CD20 nanoCAR‐T cells were generated upon treatment with both charge‐neutral and cationic LNP formulations. Depending on the transfection conditions used, nanoCAR expression could be detected up to 72 h after transfection for C12‐200‐treated T cells in the presence of ApoE. The high variability in mRNA expression kinetics observed between different biological donors is likely due to differences in T cell proliferation rates and metabolic activity. Specifically, donor T cells with a high proliferation rate exhibit short‐lived CAR expression, as both the mRNA and the expressed CAR are rapidly diluted upon cell division [77]. As the proliferative capacity is highly donor dependent, this will be an important factor to consider for effective transient cell therapies [78, 79]. Additionally, a higher initial mRNA expression per cell will prolong the persistence of mRNA expression. Previous studies have reported varying durations of mRNA‐mediated CAR expression following LNP delivery, with expression persisting from approximately 48 h up to 7 days post‐transfection [4, 24, 80, 81, 82]. However, the critical time threshold required to achieve effective therapeutic outcomes has yet to be defined. One potential strategy to extend mRNA‐based CAR expression involves reducing CAR‐T cell proliferation upon transfection. This would result in more gradual mRNA dilution as a result of lower cell division rates, thereby prolonging CAR expression [24]. In addition, the incorporation of self‐amplifying mRNA or circular mRNA could further extend mRNA expression [83, 84, 85]. These approaches maintain the inherent benefits of mRNA while optimizing expression levels and CAR persistence to support clinically effective CAR‐T cell therapies.

In addition to CAR expression on the T cell membrane, the potency of engineered T cells is of major therapeutic importance. Our results showed that both the ionizable C12‐200 and cationic DOTAP(C12‐200) LNPs allow for the engineering of transient anti‐CD20 nanoCAR‐T cells and induce effective cancer cell killing. Of note, substantial background killing was observed for mock‐transfected T cells, a phenomenon that is often not considered in the literature. As our results underscored the importance of T cell stimulation for LNP‐mediated transfection, the T cells may have been in a highly proliferative state during the potency assay. This could have facilitated secretion of cytotoxic molecules such as granzymes and perforins, potentially contributing to antigen‐independent tumor cell killing [86, 87, 88, 89]. Moreover, chronic stimulation may upregulate NK receptors on the T cell surface, which in turn could promote innate‐like, antigen‐independent responses [90]. Equally important is the influence of donor variability on nanoCAR expression and consequently, specific cell killing capacity. Overall, the presence of ApoE in the serum‐free cell culture medium improves nanoCAR expression over time for C12‐200 LNPs. Nevertheless, in some biological donors, transfection efficiency appeared less affected by ApoE, which might be caused by variability in T cell activation status during transfection. Additionally, previous research has demonstrated a positive correlation between T cell activation and LDL‐R upregulation [91]. Another study revealed that the percentage of LDL‐R^+^ T cells was directly proportional to the percentage of transduced T cells upon activation, suggesting that LDL‐R expression can serve as a predictive marker for viral transduction efficiency [61]. Hence, further investigation into the potential correlation between LDL‐R expression in activated T cells and the distinction between responders and non‐responders to ApoE‐enhanced transfection would provide valuable insights for future adoptive T cell engineering. Finally, our DOTAP(C12‐200) LNPs allowed for the engineering of functional nanoCAR‐T cells, even though lower metabolic activity was observed 24 h after LNP treatment. Nevertheless, a deeper understanding of the long‐term effects of cationic lipid‐mediated cytotoxicity on CAR‐T cell functionality will be essential for advancing their clinical translation.

## 5 Conclusion

This study demonstrates that LNP charge, cell culture medium, and T cell activation state have a profound impact on primary T cell transfection. Specifically, charge‐neutral ionizable C12‐200 LNPs showed improved transfection efficiency in the presence of recombinant ApoE protein. In contrast, our data indicates that cationic DOTAP(C12‐200) LNPs rather transfect T cells in a charge‐dependent manner and do not rely on specific proteins in the culture medium or T cell‐specific receptor expression. Overall, the use of non‐viral LNPs for ex vivo T cell engineering could represent a cost‐effective and time‐efficient alternative to current viral‐based manufacturing methods. Here, cationic LNPs offer the overall benefit of both reducing cell culture medium complexity and enabling efficient transfection across a broader T cell activation window.

## Author Contributions

L.H., K.B., and K.R. conceived the project idea and designed the experiments. K.B. and K.R. coordinated the project, supervised the research activities, and acquired funding. W.D.V. supervised the research of D.B. and acquired funding. L.H. formulated and characterized the lipid nanoparticles, performed T cell isolation, transfection experiments, functional T cell assays, and data analysis. S.D.M. assisted with the in vitro cytolytic activity assay. S.D.M, J.I., and B.V. provided anti‐CD20 nanoCAR mRNA. D.B. and M.D.V. assisted with T cell isolation. M.D.V. contributed to flow cytometry analysis. L.H. wrote the article and prepared the figures. All authors contributed to manuscript reviewing and editing.

## Conflicts of Interest

The authors declare no conflicts of interest.

## Supporting information




**Supporting File**: advs74482‐sup‐0001‐SuppMat.docx.

## Data Availability

The data that support the findings of this study are available from the corresponding author upon reasonable request.
