# Supplementary material for: Cationic mRNA Lipid Nanoparticles for Ex Vivo NanoCAR‐T Cell Engineering
Source: Adv Sci (Weinh). 2026 Mar 24;13(24):e21507. doi: 10.1002/advs.202521507 (PMC13116164; doi:10.1002/advs.202521507)
Supplement: Supplementary file 1 — Supporting File: advs74482‐sup‐0001‐SuppMat.docx. [file ADVS-13-e21507-s001.docx]

Supporting Information

Cationic mRNA Lipid Nanoparticles for

Ex Vivo NanoCAR-T Cell Engineering

*Laure Harinck^1,4^, Stijn De Munter^2,4^, Margo De Velder^1,4^, Joline Ingels^3,4^, Dominika Berdecka^1^, Ine Lentacker^1,4^, Winnok H. De Vos^5,6^, Bart Vandekerckhove^2,3,4^, Kevin Braeckmans^1,4^, Koen Raemdonck^1,4,*^*

^1^ Laboratory for General Biochemistry and Physical Pharmacy, Faculty of Pharmaceutical Sciences, Ghent University, Ghent, Belgium

^2^ Department of Diagnostic Sciences, Faculty of Medicine and Health, Ghent University, Ghent, Belgium

^3^ GMP Unit Cell & Gene Therapy, Ghent University Hospital, Ghent, Belgium

^4^ Cancer Research Institute Ghent (CRIG), Ghent, Belgium

^5^ Cell Biology and Histology Lab, University of Antwerp, Antwerp, Belgium

^6^ Antwerp Centre for Advanced Microscopy, Antwerp, Belgium

***Author e-mail addresses****:* [*laure.harinck@UGent.be*](mailto:laure.harinck@UGent.be)*,* [*stijn.demunter@UGent.be*](mailto:stijn.demunter@UGent.be)*,* [*joline.ingels@uzgent.be*](mailto:joline.ingels@uzgent.be)*,* [*margo.develder@UGent.be*](mailto:margo.develder@UGent.be)*,* [*dominika.berdecka@UGent.be*](mailto:dominika.berdecka@UGent.be)*,* [*winnok.devos@uantwerpen.be*](mailto:winnok.devos@uantwerpen.be)*,* [*bart.vandekerckhove@UGent.be*](mailto:bart.vandekerckhove@UGent.be)*,* [*kevin.braeckmans@UGent.be*](mailto:kevin.braeckmans@UGent.be)*,* [*koen.raemdonck@UGent.be*](mailto:koen.raemdonck@UGent.be)

* Corresponding author: Prof. Koen Raemdonck, E-mail: [koen.raemdonck@UGent.be](mailto:koen.raemdonck@UGent.be)

**Keywords**: Lipid nanoparticles, mRNA delivery, CAR-T cells, Non-viral gene delivery, Immunotherapy, Cell engineering, Nanomedicine.

**Table S1. Amino acid sequence of proteins used in this study.**

Human Apolipoprotein E – ApoE3 protein, His Tag: Target – Linker(gggsgggs) – 10*His(hhhhhhhhhh)

KVEQAVETEPEPELRQQTEWQSGQRWELALGRFWDYLRWVQTLSEQVQEELLSSQVTQELRALMDETMKELKAYKSELE

QLTPVAEETRARLSKELQAAQARLGADMEDVCGRLVQYRGEVQAMLGQSTEELRVRLASHLRKLRKRLLRDADDLQKRLA

VYQAGAREGAERGLSAIRERLGPLVEQGRVRAATVGSLAGQPLQERAQAWGERLRARMEEMGSRTRDRLDEVKEQVAEVR

AKLEEQAQQIRLQAEAFQARLKSWFEPLVEDMQRQWAGLVEKVQAAVGTSAAPVPSDNH

Human Transferrin Protein, His Tag (MALS verified): Target – Linker(gggsgggs) – 10*His(hhhhhhhhhh)

VPDKTVRWCAVSEHEATKCQSFRDHMKSVIPSDGPSVACVKKASYLDCIRAIAANEADAVTLDAGLVYDAYLAPNNLKPV

VAEFYGSKEDPQTFYYAVAVVKKDSGFQMNQLRGKKSCHTGLGRSAGWNIPIGLLYCDLPEPRKPLEKAVANFFSGSCAP

CADGTDFPQLCQLCPGCGCSTLNQYFGYSGAFKCLKNGAGDVAFVKHSTIFENLANKADRDQYELLCLDNTRKPVDEYKD

CHLAQVPSHTVVARSMGGKEDLIWELLNQAQEHFGKDKSKEFQLFSSPHGKDLLFKDSAHGFLKVPPRMDAKMYLGYEYV

TAIRNLREGTCQEAPTDECKPVKWCALSHHERLKCDEWSVNSVGKIECVSAETTEDCIAKIMNGEADAMSLDGGFVYIAG

KCGLVPVLAENYNKSDNCEDTPEAGYFAVAVVKKSASDLTWDNLKGKKSCHTAVGRTAGWNIPMGLLYNKINHCRFDEFF

SEGCAPGSKKDSSLCKLCMGSGLNLCEPNNKEGYYGYTGAFRCLVEKGDVAFVKHQTVPQNTGGKNPDPWAKNLNEKDYE

LLCLDGTRKPVEEYANCHLARAPNHAVVTRKDKEACVHKILRQQQHLFGSNVTDCSGNFCLFRSETKDLLFRDDTVCLAK

LHDRNTYEKYLGEEYVKAVGNLRKCSTSSLLEACTFRRP

**Table S2. Encapsulation efficiency (EE) and final encapsulated mRNA concentration (ng/µL) as determined using a Ribogreen assay.** Data represent mean ± SD of C12-200 LNPs encapsulating enhanced green fluorescent protein (eGFP)-encoding mRNA (*n* = 24), C12-200 LNPs encapsulating firefly luciferase (Luc)-encoding mRNA (*n* = 16), DOTAP(C12-200) eGFP LNPs (*n* = 12), DOTAP(C12-200) Luc LNPs (*n* = 7), with *n* representing independent LNP formulations.

| LNP | EE (%) | Final encapsulated concentration mRNA (ng/µL) |
| --- | --- | --- |
| C12-200 eGFP | 98.4 ± 1.4 | 106.7 ± 18.7 |
| C12-200 Luc | 98.6 ± 1.5 | 106.8 ± 19.0 |
| DOTAP(C12-200) eGFP | 98.8 ± 1.3 | 99.0 ± 9.8 |
| DOTAP(C12-200) Luc | 98.7 ± 1.3 | 110.2 ± 10.3 |


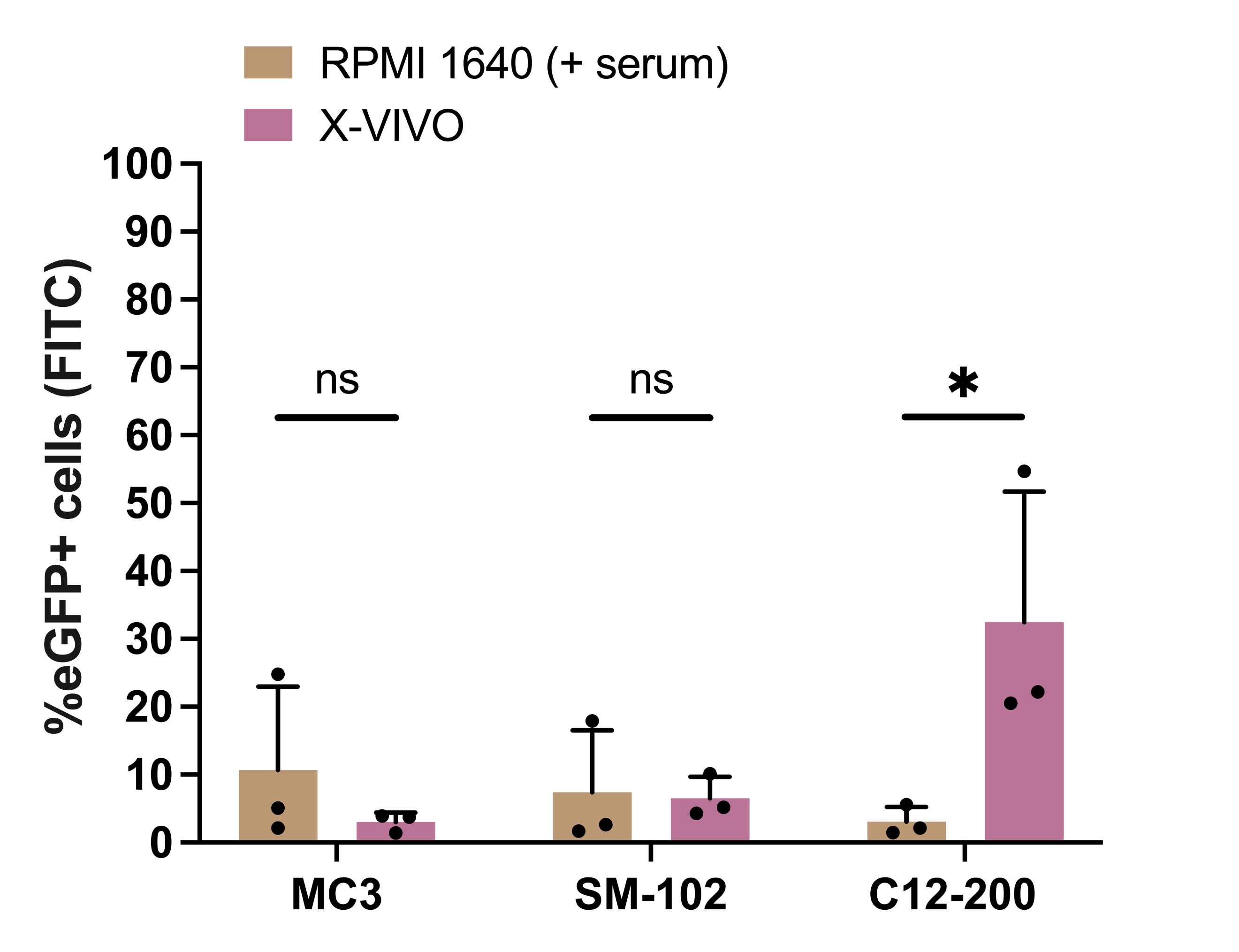


**Figure S1. Transfection of Jurkat cells with ionizable LNPs in different cell culture media.** eGFP mRNA lipid nanoparticle (LNP) formulations with varying ionizable lipids used for transfection of Jurkat cell line in different cell culture media with or without additional FBS (RPMI + 10% FBS (Fetal Bovine Serum) vs X-VIVO). All LNPs were formulated at molar ratios of 50:10:38.5:1.5 (ionizable lipid:DOPE:cholesterol:DMG-PEG). Transfection data are presented as mean ± SD (*n* = 3, biological replicates). Statistical analysis was performed using ordinary Two-way ANOVA with Šidák’s multiple comparisons test (ns *p*>0.05; **p*<0.05; ***p*<0.01; ****p*<0.001; *****p*<0.0001).


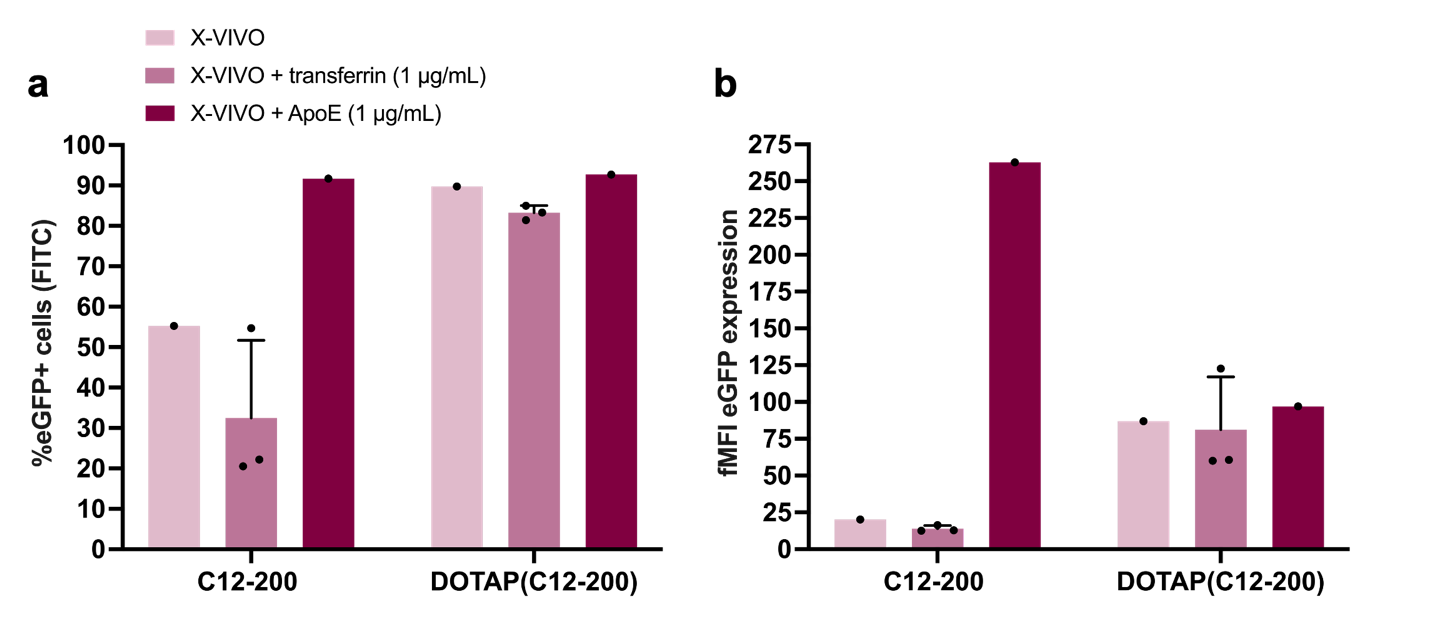


**Figure S2. Influence of extracellular proteins in serum-free X-VIVO transfection medium on transfection of Jurkat cells.** Transfection efficiency (% eGFP+ cells) and fold mean fluorescence intensity (fMFI) values of Jurkat cells after 24 h incubation of 1 ng/µL eGFP mRNA lipid nanoparticles (LNPs) in serum-free X-VIVO or X-VIVO spiked with respectively transferrin (1 µg/mL) and apolipoprotein E (ApoE) protein (1 µg/mL). Data represent mean ± SD (X-VIVO and X-VIVO + ApoE: *n* = 1; X-VIVO + transferrin: *n* = 3, biological replicates).


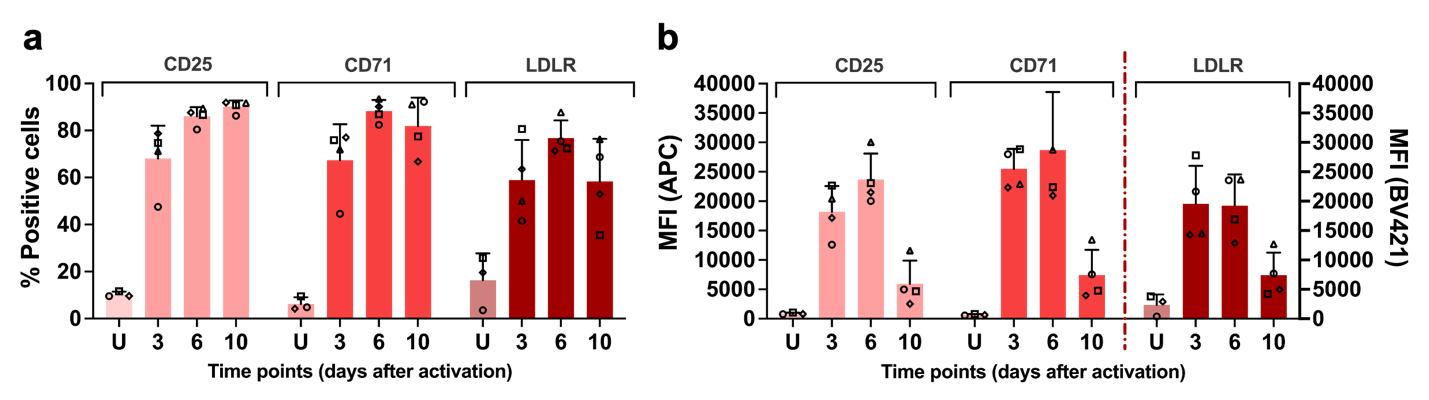


**Figure S3. Receptor expression over time on activated T cells.** Primary human T cells were stimulated using ImmunoCult CD3/CD28/CD2 activator on the day of isolation and kept in culture for up to 10 days at a concentration of 1 x 10^6^ cells/mL. **(a)** Receptor expression of CD25 as activation marker, CD71 as transferrin receptor, and LDL-R (low-density lipoprotein receptor) as ApoE (apolipoprotein E) receptor were evaluated by flow cytometry on day 0 (unstimulated T cells, U), day 3, day 6, day 10 (activated T cells). Data represents the percentage of cells positive for CD25, CD71, and LDL-R, respectively. **(b)** Mean fluorescence intensity (MFI) of stimulated T cells as an indication of receptor intensity after activation. Data are presented as mean ± SD of four different donors (*n* = 4, each represented by a different symbol).


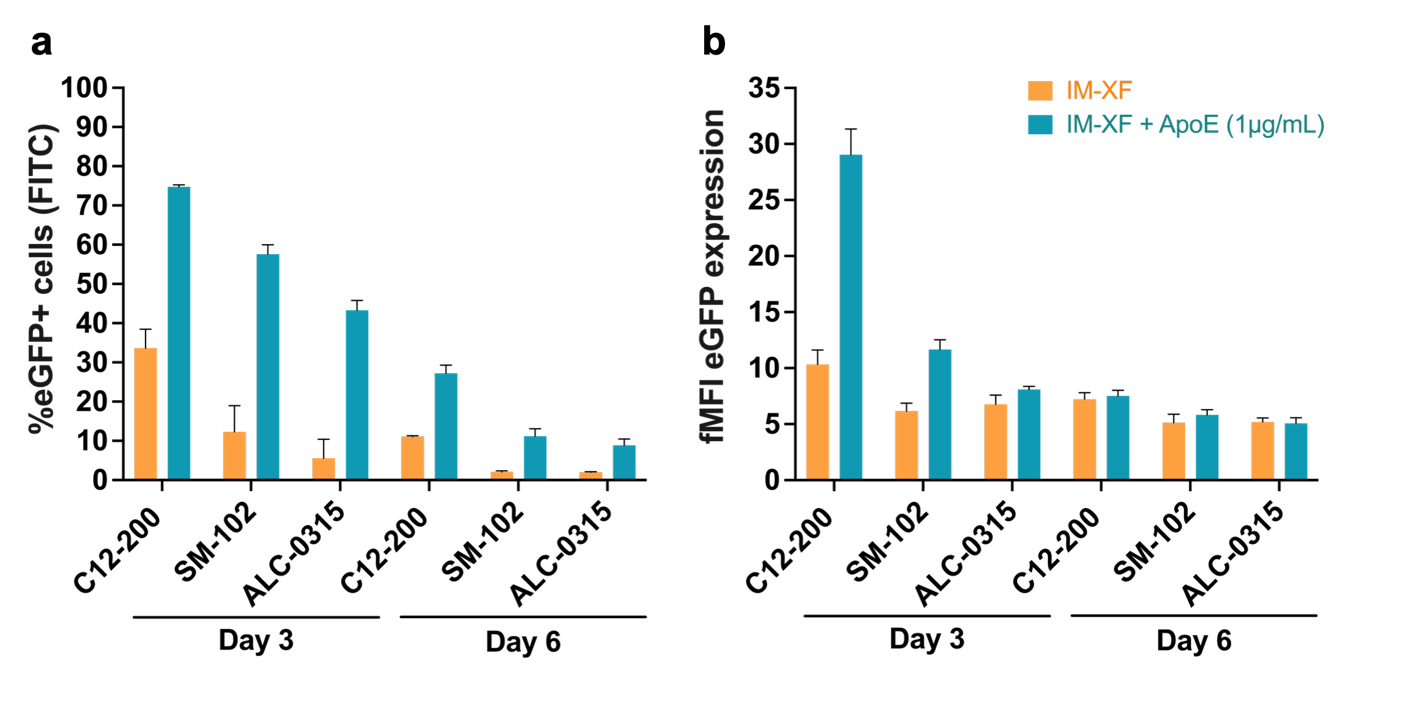


**Figure S4. Influence of ionizable lipids on T cell transfection. (a)** Transfection efficiency and **(b)** fold mean fluorescence intensity (fMFI) of activated CD3^+^ T cells determined with flow cytometry. T cells were transfected on day 3 or day 6 after initial stimulation (= day 0) using lipid nanoparticle (LNP) formulations containing distinct ionizable lipids (i.e., C12-200, SM-102, and ALC-0315). A dose of 1 ng/µL encapsulated eGFP mRNA was added in both ImmunoCult™-XF T Cell expansion medium (IM-XF) and IM-XF + apolipoprotein E (ApoE) (1 µg/mL). Data are presented as mean ± SD (*n* = 3, technical replicates for one biological donor).


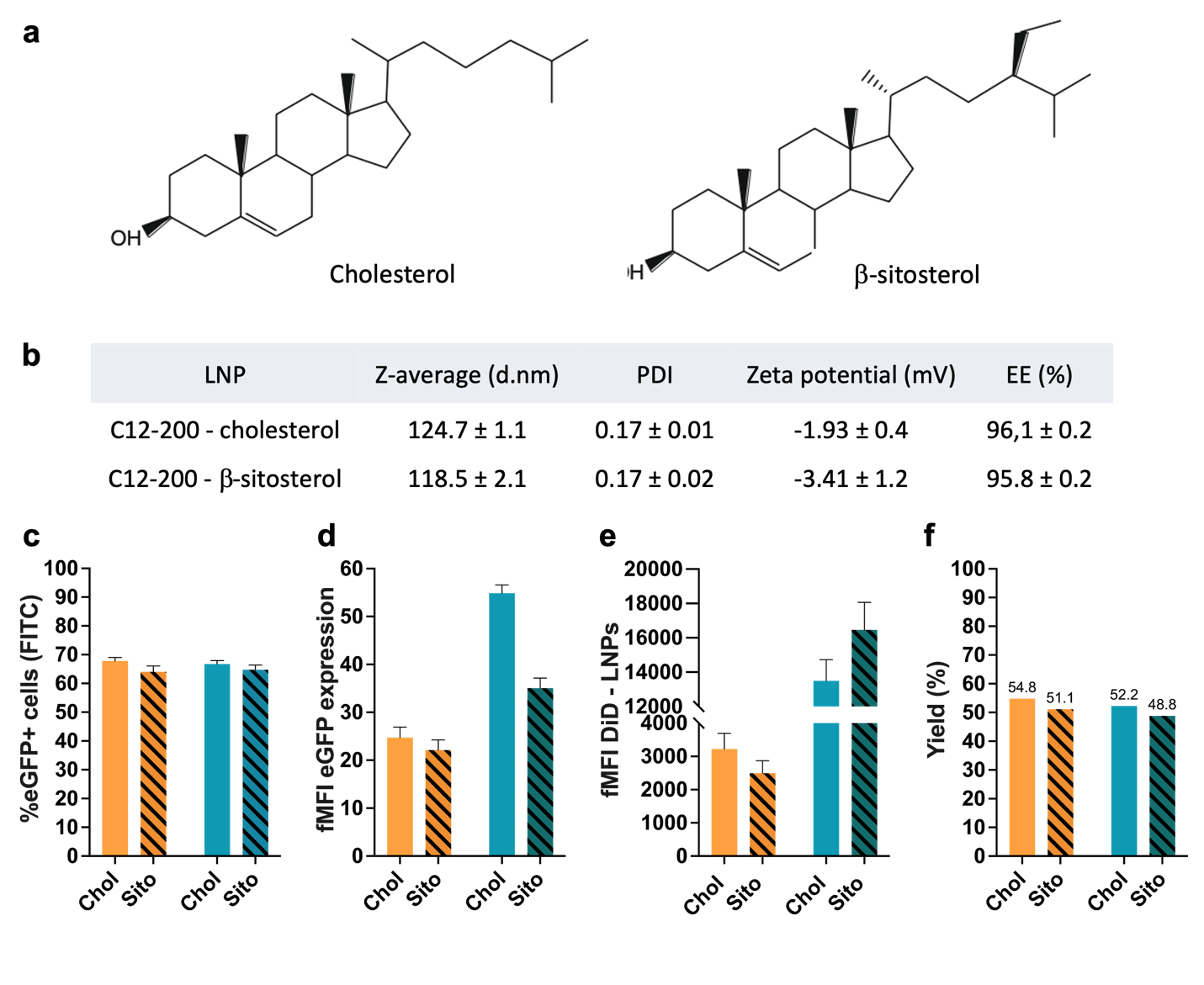


**Figure S5. Substitution of cholesterol by β-sitosterol to optimize LNP formulations. (a)** Schematic representation of cholesterol analogues. **(b)** Physicochemical characterization of ionizable lipid nanoparticles (LNPs) containing cholesterol and β-sitosterol, respectively. **(c)** Transfection efficiency and **(d)** fold mean fluorescence intensity (fMFI) of primary human T cells transfected with 3 ng/µL eGFP mRNA encapsulated in C12-200(cholesterol) or C12-200(b-sitosterol) LNPs. Transfection was performed on day 3 after initial stimulation in both ImmunoCult™-XF T Cell expansion medium (IM-XF) and IM-XF + apolipoprotein E (ApoE) (1 µg/mL). **(e)** Representation of LNP-T cell interaction by evaluation of fMFI of DiD-labeled LNPs. **(f)** Percentage of viable transfected cells, displayed as yield (%). Data are presented as mean ± SD (*n* = 3, technical replicates for 1 biological donor). Abbreviations: PDI, Polydispersity index; EE, encapsulation efficiency.


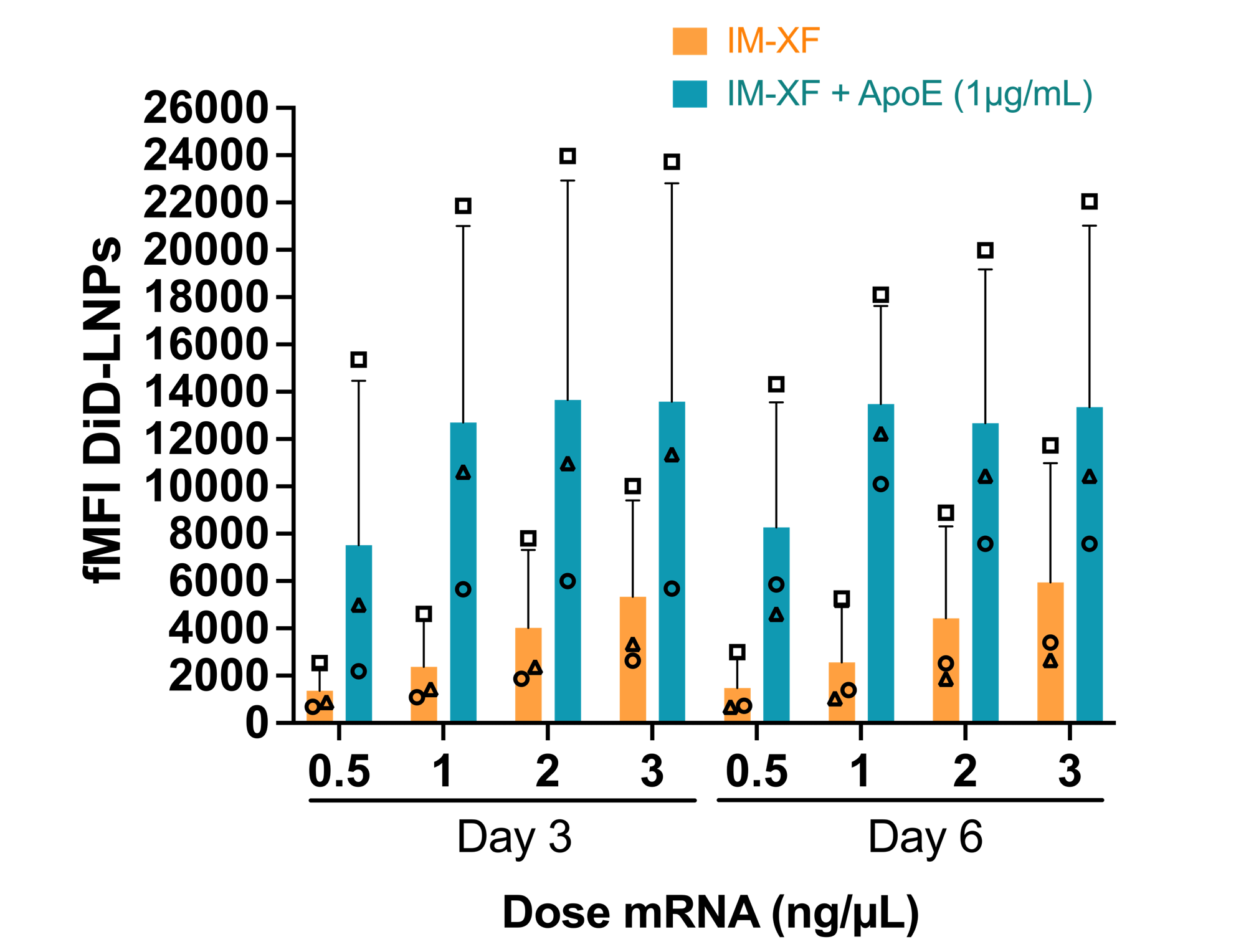


**Figure S6. LNP interaction with primary human T cells as a function of mRNA LNP dose.** T cells were transfected in ImmunoCult™-XF T Cell expansion medium (IM-XF) or IM-XF + apolipoprotein E (ApoE) (1 µg/mL) on day 3 or 6 post-activation using C12-200 lipid nanoparticles (LNPs) containing 1.2 mol% DiD-lipid dye at an mRNA dose of 0.5 to 3 ng/µL for 5 x 10^4^ T cells. After 24 h incubation, fold mean fluorescence intensity (fMFI) of DiD-LNPs was determined with flow cytometry for the evaluation of LNP-T cell interaction. fMFI was calculated as MFI DiD+ cells/MFI Luc CTR cells. Data represent mean ± SD with individual donors represented by different symbols (*n* = 3, biological independent donors).

**
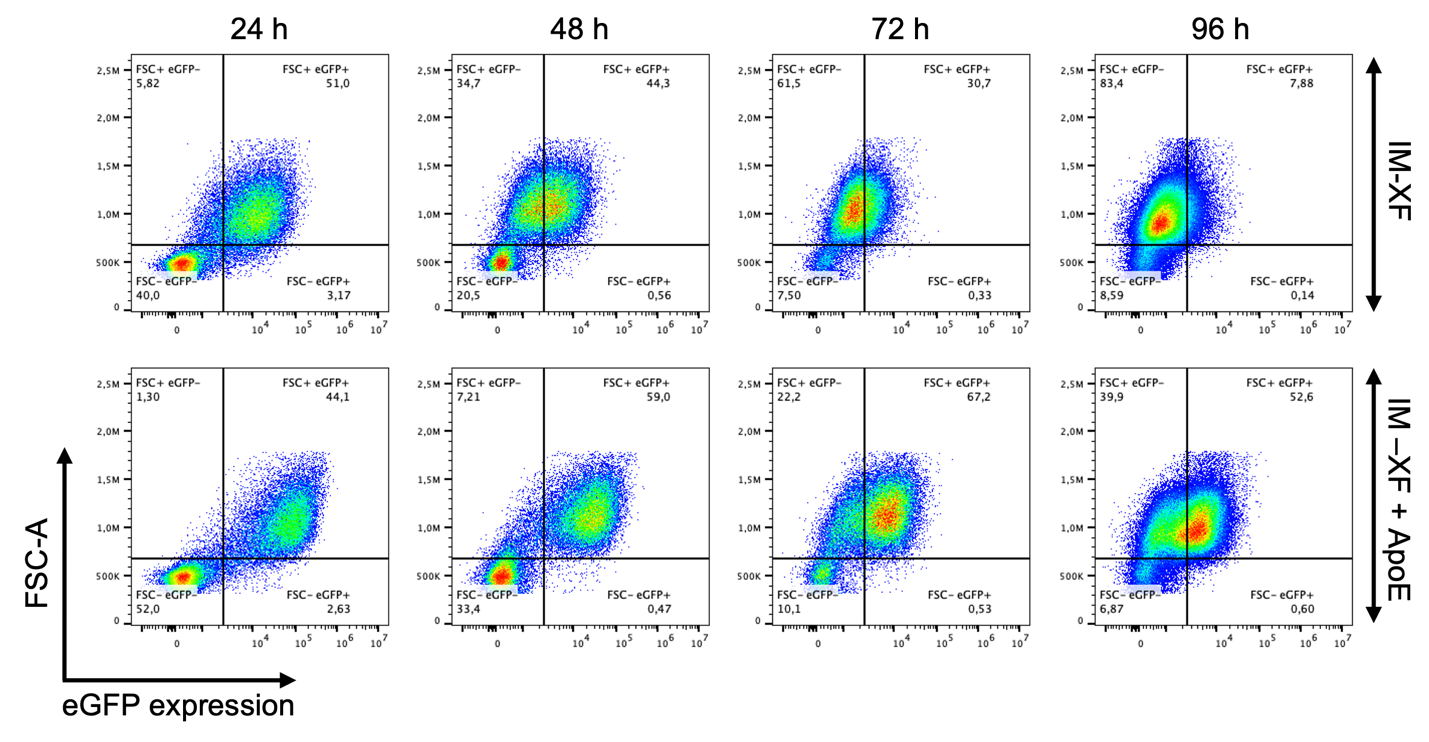
**

**Figure S7. Flow cytometry gating strategy used to determine kinetics of eGFP protein expression over time in CD3^+^ stimulated T cells.** Primary human T cells were isolated and activated (day 0) with ImmunoCult CD3/CD28/CD2 activator. On day 3 after initial activation, cells were transfected using C12-200 lipid nanoparticles (LNPs) at a dose of 3 ng/µL encapsulated eGFP mRNA. Transfections were performed in both ImmunoCult™-XF T Cell expansion medium (IM-XF) and IM-XF + apolipoprotein E (ApoE) (1 µg/mL) and eGFP protein expression was determined on fixed time points (i.e., 24 h, 48 h, 72 h, and 96 h) after transfection with flow cytometry. Cells were gated on difference in forward scatter area (FSC-A) to distinguish between FSC_high_ and FSC_low_ cells. eGFP positive cells were determined based on their FITC-area signal. Gatings have been set against C12-200 Luc mRNA LNP-treated T cells with similar transfection conditions.


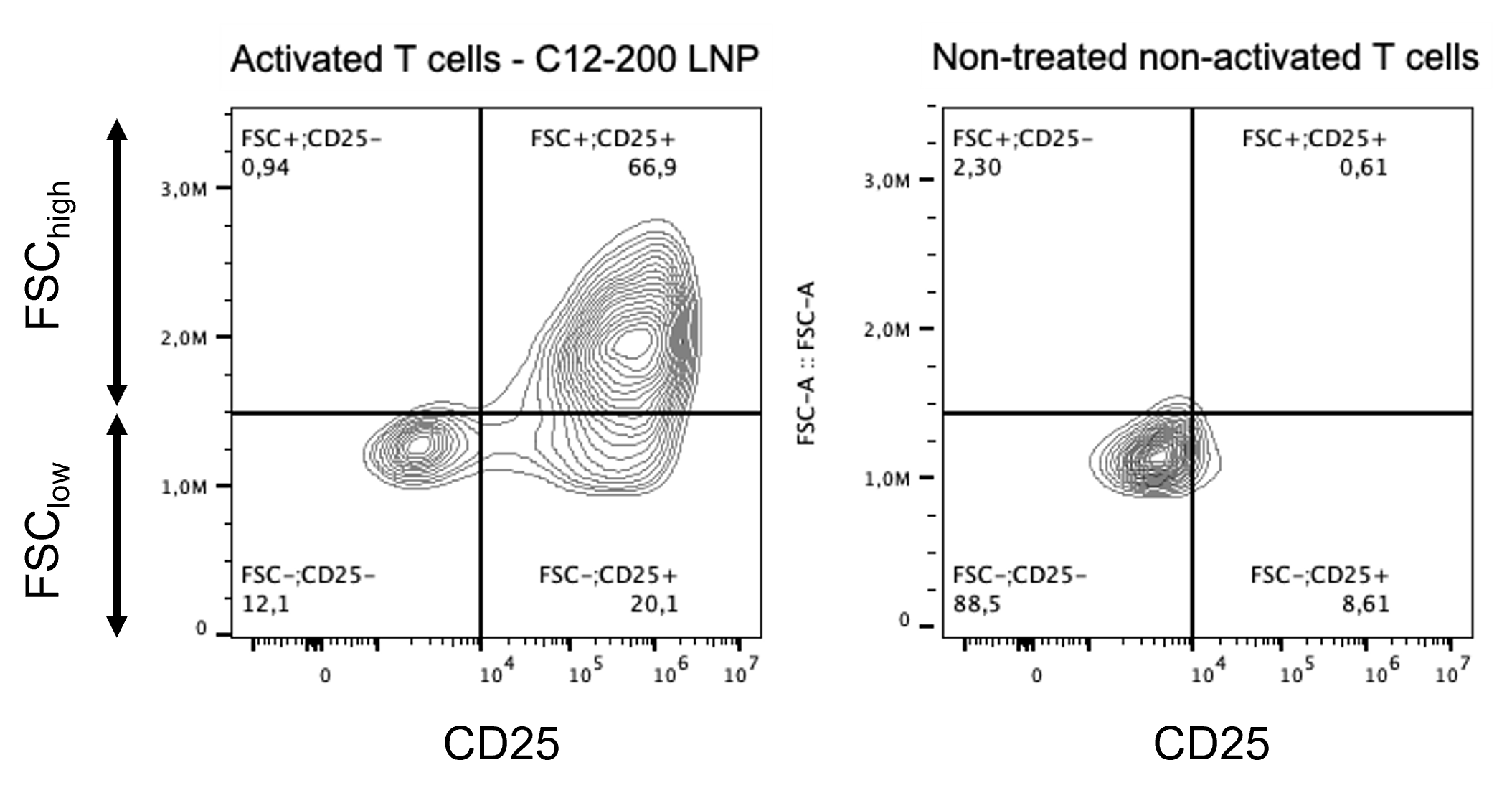


**Figure S8.** Flow cytometry plots of (left) CD3+ T cells labeled with CD25-APC antibody on day 3 after activation and following C12-200 LNP transfection and (right) non-treated and non-activated CD3+ T cells labeled with CD25-APC antibody. Gating was set to distinguish between forward scatter high (FSC_high_) and FSC_low_ T cell populations.

**
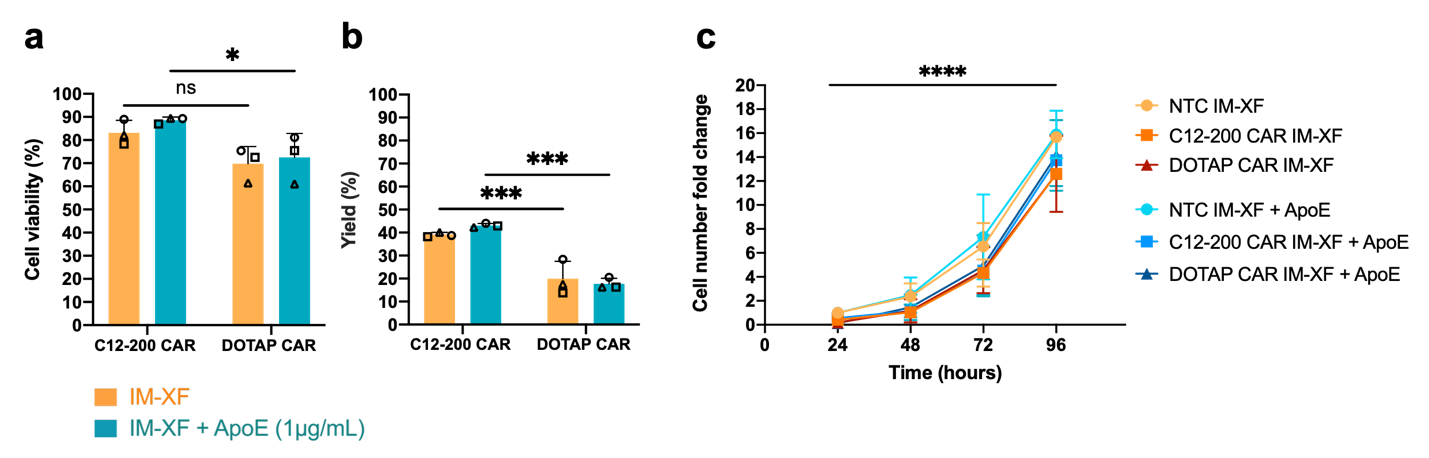
**

**Figure S9. Cell viability, yield and cell growth kinetics after ionizable and cationic LNP treatment on activated T cells.** **(a)** Cell viability of activated primary human T cells determined by CellTiter-Glo® assay after 24 h incubation with C12-200 or DOTAP(C12-200) lipid nanoparticles (LNPs) encapsulating anti-CD20 nanoCAR mRNA at a dose of 3 ng/µL and 1 ng/µL, respectively. T cells were transfected on day 3 after initial activation. **(b)** Yield, or percentage of viable transfected cells, after 24 h incubation. Results obtained by multiplying % nanoCAR+ cells (total population) by corresponding cell viability (%). Statistics were performed using repeated measures Two-way ANOVA with LNP treatment and media as within-subject factors. Data represents three independent donors (*n* = 3, indicated as circle, triangle, square), with matched values for each donor spread across rows. Šidák’s multiple comparisons test with single pooled variance was used for post-hoc comparisons between LNP treatments. Error bars represent mean ± SD. **(c)** Cell number fold change in T cell cultures treated with LNPs in ImmunoCult™-XF T Cell expansion medium (IM-XF) and IM-XF + Apolipoprotein E (ApoE) (1 µg/mL). Absolute cell counts were assessed by flow cytometry, and cell growth was normalized to cell count of untreated cells (NTC) at 24 h in the respective media. Statistical analysis was performed by a mixed-effects model to account for repeated measurements, with time and LNP treatment as fixed effects and donor variability as random effect. Tukey’s multiple comparison test with single pooled variance was used to perform post-hoc comparisons between time points. Significant differences are indicated as follows: ns *p*>0.05; **p*<0.05; ***p*<0.01; ****p*<0.001; *****p*<0.0001. Error bars represent mean ± SD, *n* = 3 biological independent donors.


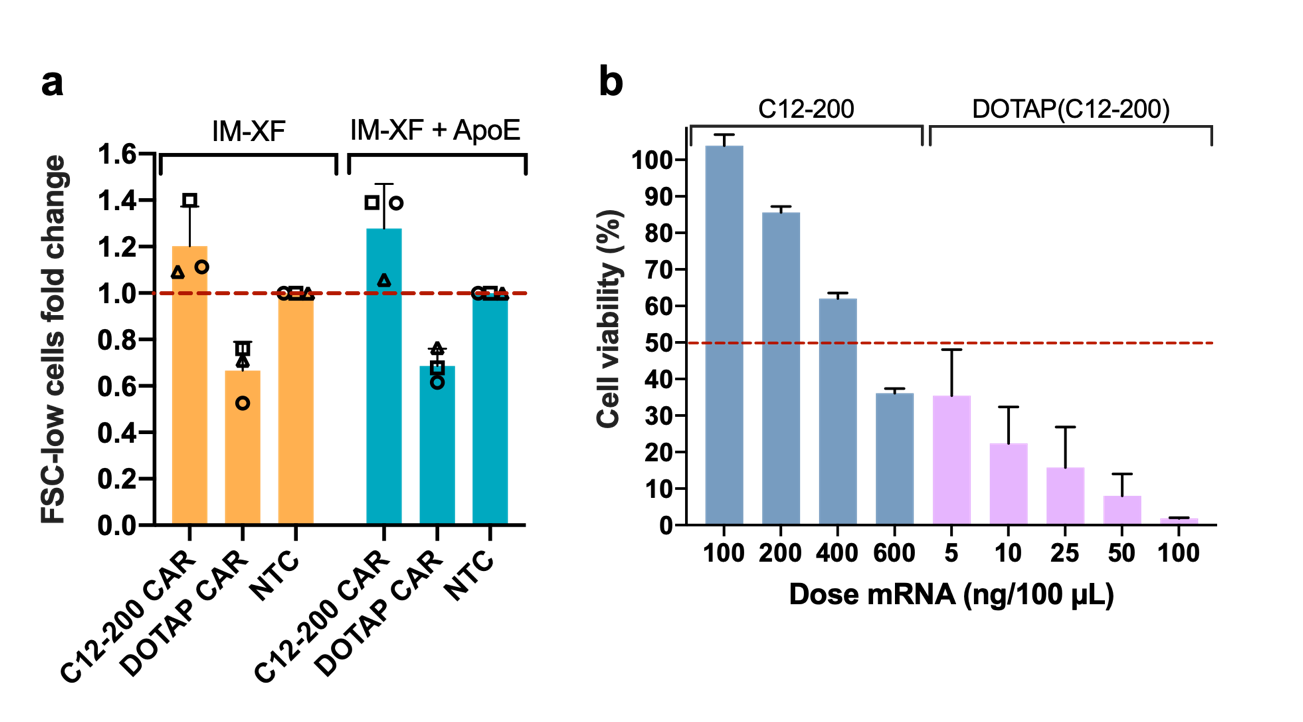


**Figure S10. Toxicity on unstimulated T cells induced by cationic DOTAP(C12-200) LNPs.** **(a)** Transfection of activated T cells on day 3 after isolation and activation using ionizable C12-200 or cationic DOTAP(C12-200) nanoCAR mRNA lipid nanoparticles (LNPs) at a dose of 3 ng/µL and 1 ng/µL, respectively. Determination of forward scatter low (FSC_low_) cell population after 24 h incubation with LNPs was performed using flow cytometry. FSC_low_ cells fold change was calculated by normalization of % FSC_low_ cells after LNP treatment to % FSC_low_ cells of untreated condition (NTC). Data represent mean ± SD, *n* = 3 independent biological donors. **(b)** Cell viability of unstimulated T cells after 24 h incubation with C12-200 or DOTAP(C12-200) LNPs was determined with CellTiter-Glo® assay. Varying doses of encapsulated eGFP mRNA have been tested. Data represent mean ± SD, *n* = 3 technical replicates for one biological donor.


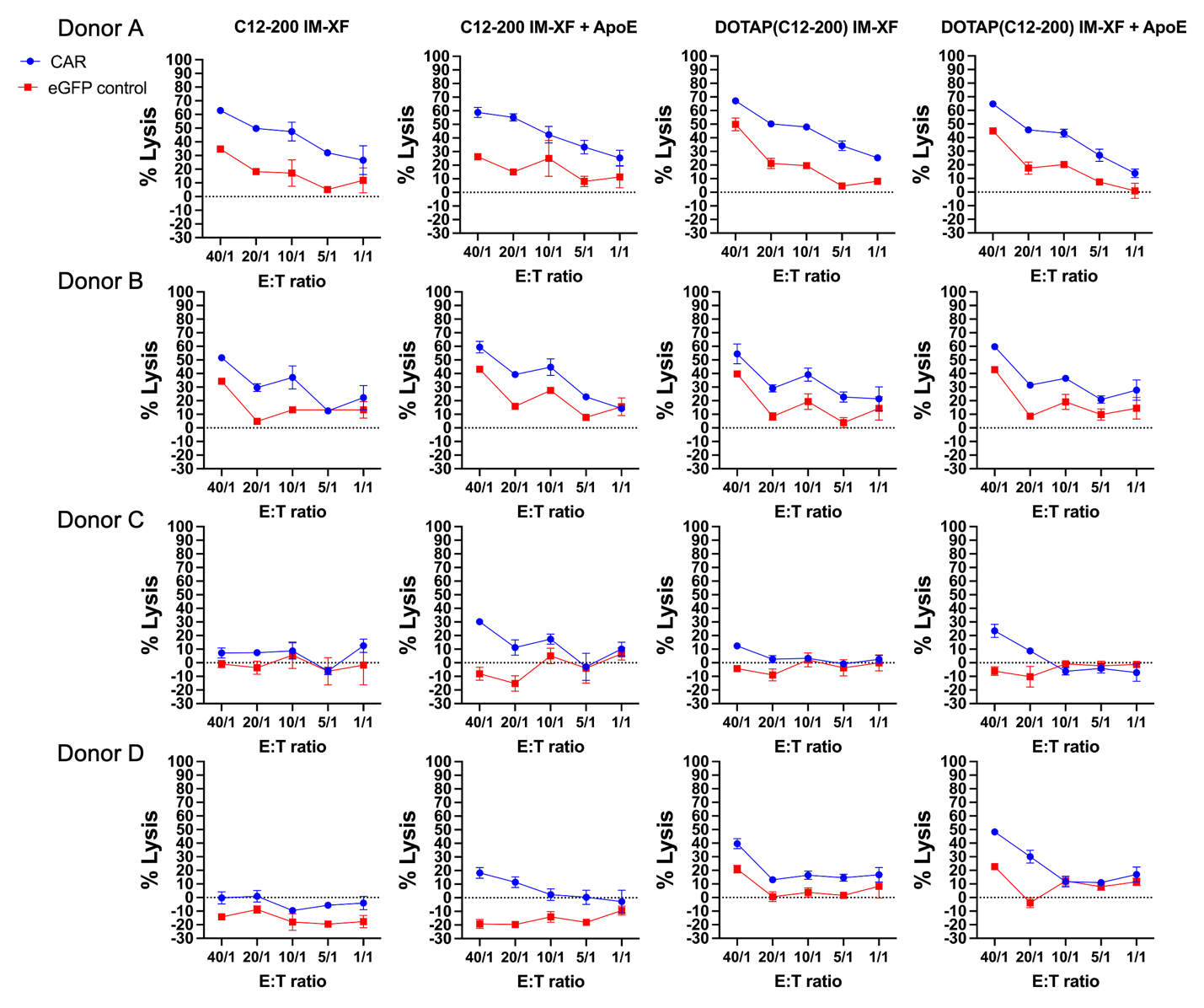


**Figure S11. Cytolytic activity of anti-CD20 nanoCAR-T cells and eGFP mRNA treated control T cells against CD20^+^ Raji cell line.** Cell lysis (%) of CD20^+^ Raji target cells expressing luciferase after 24 h co-culture with lipid nanoparticle (LNP)-mediated engineered anti-CD20 nanoCAR-T cells in different effector-to-target (E:T) ratios. C12-200 and DOTAP(C12-200) LNPs, encapsulating anti-CD20 nanoCAR mRNA or eGFP mRNA, were used for engineering of activated primary human T cells (day 3 after stimulation) by 24 h incubation in ImmunoCult™-XF T Cell expansion medium (IM-XF) or IM-XF + apolipoprotein E (ApoE) (1 µg/mL) medium. The Raji cell lysis (%) was determined 48 h post T cell transfection. eGFP mRNA LNPs were used as control treatment to determine unspecific background lysis. Reported data represent mean ± standard error of the mean (SEM) (*n* = 3, technical replicates). Results shown for two independent experiments, performed with four different donors (i.e., Donor A, B, C, and D).


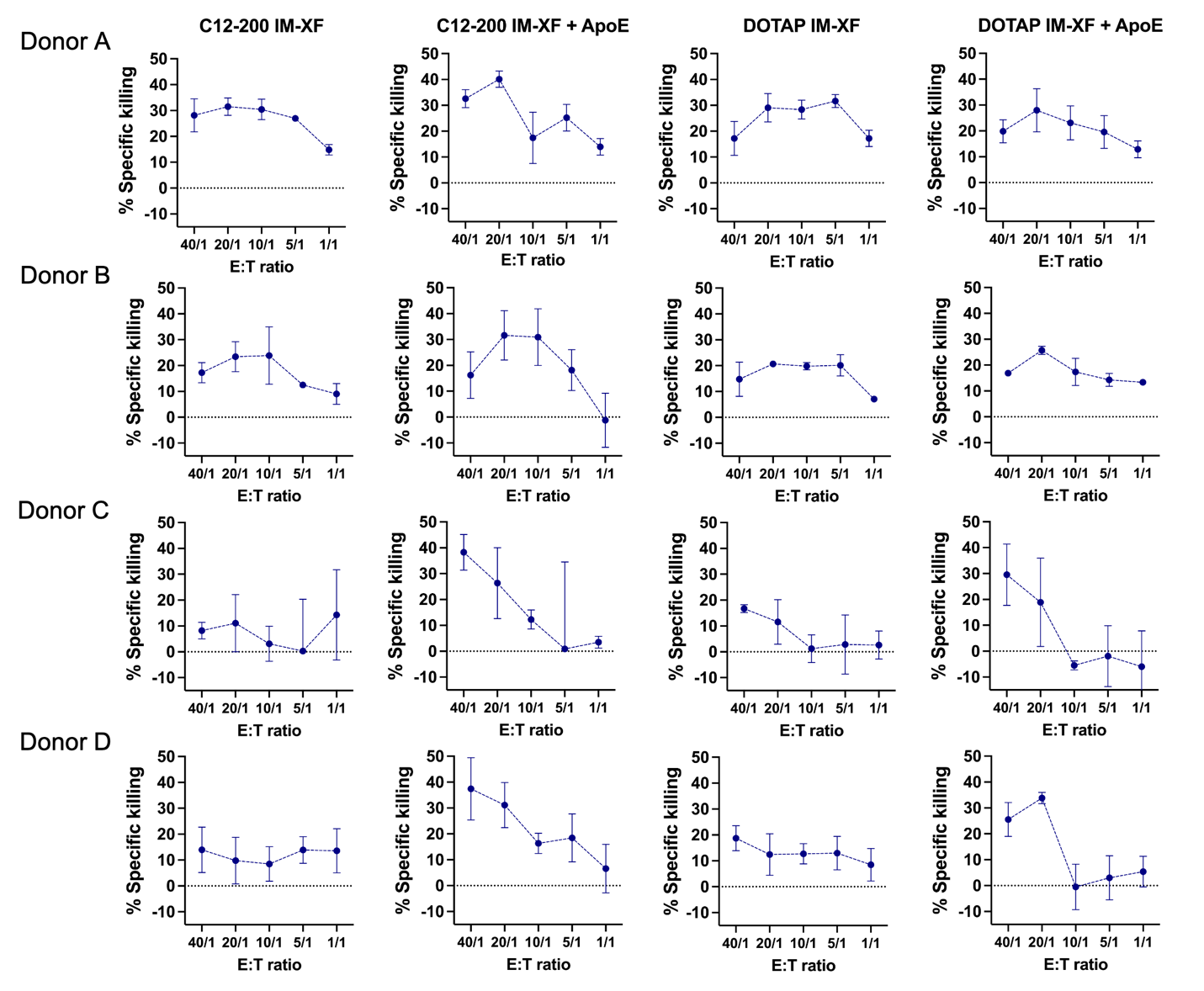


**Figure S12. Specific cytotoxicity of anti-CD20 nanoCAR-T cells against luciferase expressing Raji cell line.**  Specific cell lysis (%) of CD20^+^ Raji target cells expressing luciferase after 24 h incubation with T cells expressing anti-CD20 nanoCAR in different effector-target (E:T) ratios. Anti-CD20 nanoCAR-T cells were engineered by incubation of C12-200 and DOTAP(C12-200) lipid nanoparticles (LNPs), encapsulating anti-CD20 nanoCAR mRNA, for 24 h in both ImmunoCult™-XF T Cell expansion medium (IM-XF) and IM-XF + apolipoprotein E (ApoE) (1 µg/mL). Specific CAR-T mediated cell lysis was determined 48 h post-transfection. eGFP mRNA LNPs were used as control treatment and a correction for unspecific background lysis was performed. Reported data represent mean ± standard error of the mean (SEM) (*n* = 3, technical replicates). Data represents two independent experiments, performed with four different donors (i.e., Donor A, B, C, and D).


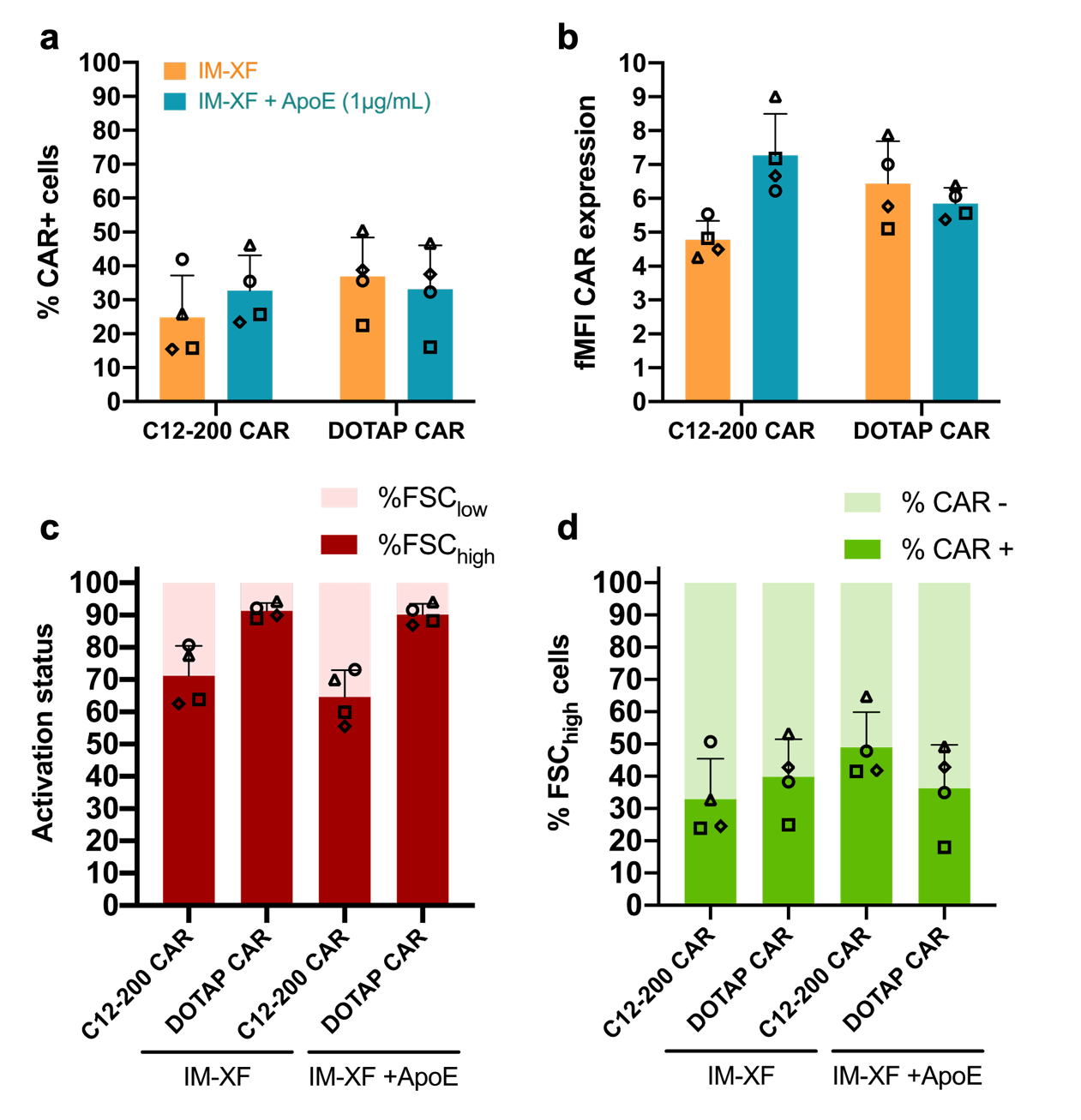


**Figure S13. CAR expression and activation status for biological donors used in Raji cell killing experiment.** Anti-CD20 nanoCAR expression in primary human T cells determined after treatment with respectively C12-200 (3 ng/µL mRNA) and DOTAP(C12-200) lipid nanoparticles (LNPs) (1 ng/µL mRNA) in both ImmunoCult™-XF T Cell expansion medium (IM-XF) and IM-XF + apolipoprotein E (ApoE) (1 µg/mL). Transfection was performed on activated T cells (day 3 after initial stimulation) and anti-CD20 nanoCAR expression was assessed after 24 h LNP incubation. Flow cytometry was used to determine **(a)** % nanoCAR+ cells in total T cell population, **(b)** fold median fluorescence intensity (fMFI), **(c)** Activation status by distinction between forward scatter high (FSC_high_) and FSC_low_ population. **(d)** Percentage of nanoCAR+ FSC_high_ cells represented as the ratio of % nanoCAR+ cells in FSC_high_ population divided by % FSC_high_ cells of total population. Data represents mean ± SD of 4 biological independent donors (*n* = 4, each donor depicted by a different symbol).

**
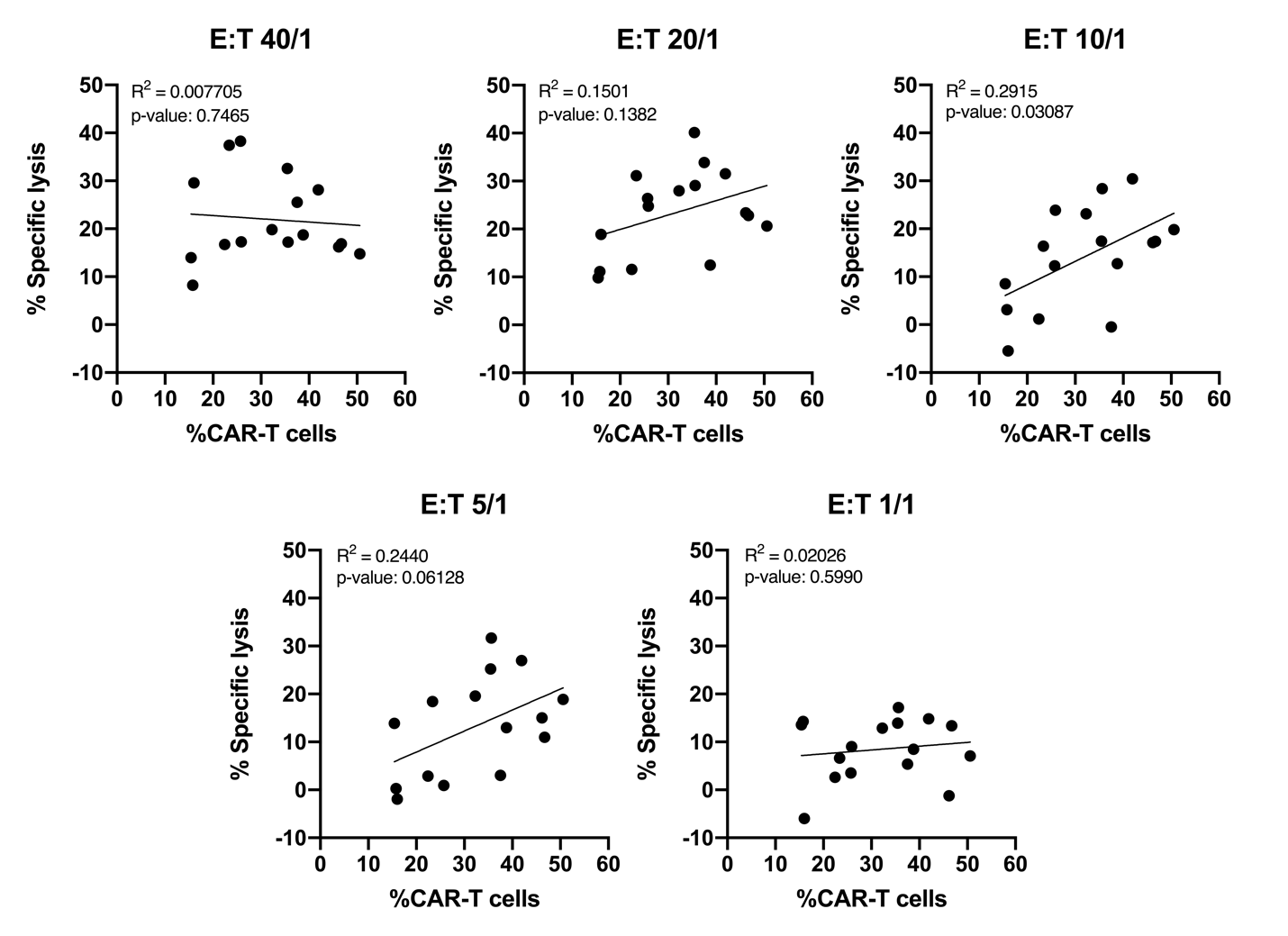
**

**Figure S14. Correlation between % nanoCAR-T cells and % specific lysis of Raji cells for different E:T ratios.** Scatterplots show the relationship between percentage engineered anti-CD20 nanoCAR-T cells and specific cytolytic activity (%) against CD20^+^ Raji cell line for effector-to-target (E:T) ratios 40/1, 20/1, 10/1 and 5/1, respectively. All treatment conditions were combined (i.e., C12-200 and DOTAP(C12-200) lipid nanoparticle (LNP)-treated T cells in both ImmunoCult™-XF T Cell expansion medium (IM-XF) and IM-XF + apolipoprotein E (ApoE) (1 µg/mL)) and a correlation plot with simple linear regression was performed. No significant correlation was observed for E:T ratio of 40/1 and 1/1 (*r* = -0.088, *p* = 0.75, *R^2^* = 0.0077 and *r* = 0.14, *p* = 0.60, *R^2^* = 0.020, respectively). There was a trend toward significance (*r* =0.39, *p* = 0.14, *R^2^* = 0.15 and *r* = 0.49, *p* = 0.06, *R^2^* = 0.24) for E:T ratios 20/1 and 5/1, respectively. A significant positive correlation was observed for E:T ratio 10/1 (*r* = 0.54, *p* = 0.03, *R^2^* = 0.29). The solid line represents the best-fit linear regression (Y = 0.4883X - 1.450). Data represents four biological independent donors (*n* = 4).


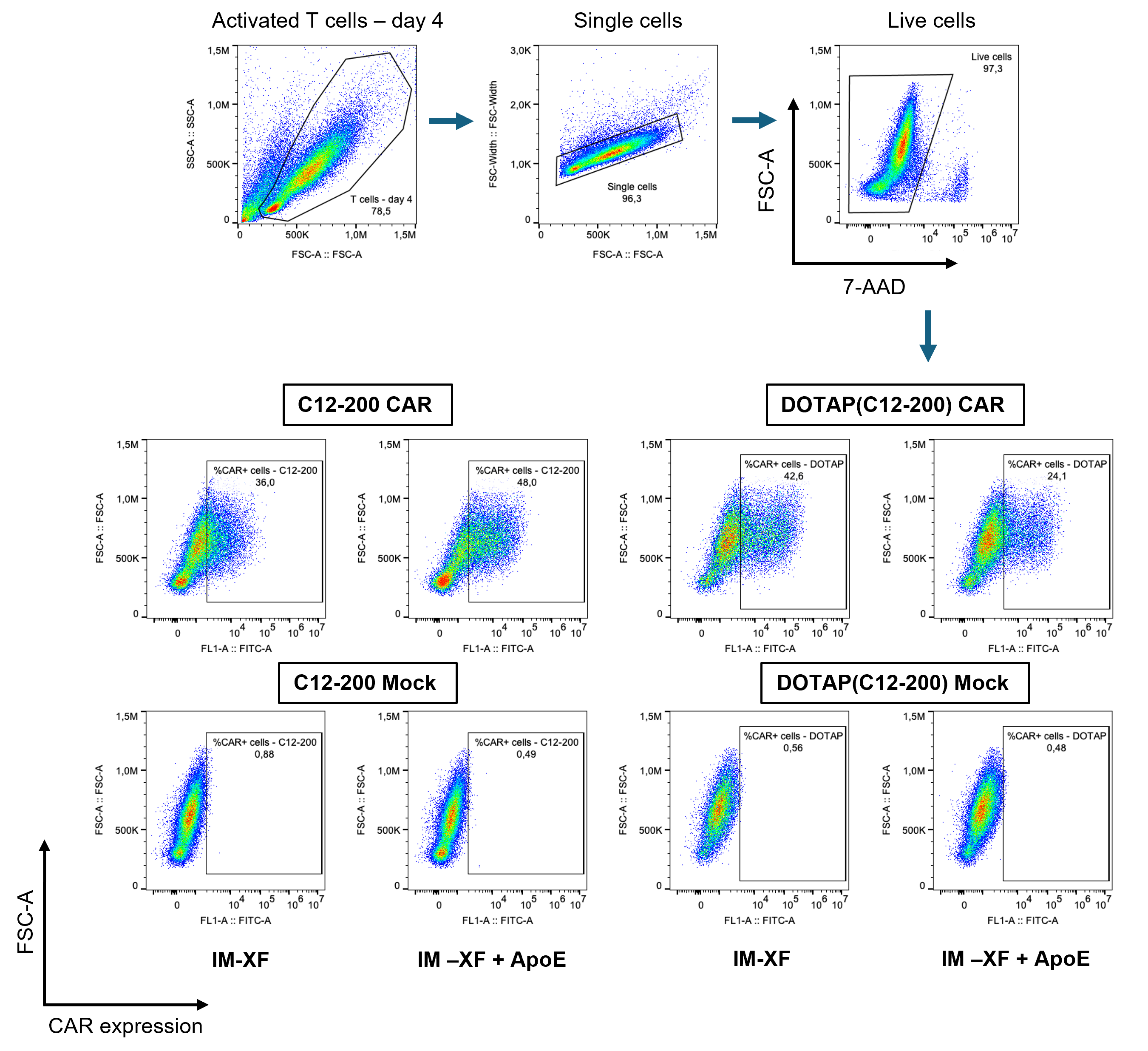


**Figure S15. Representative flow cytometry plots and gating strategy to quantify nanoCAR+ primary human T cells.** Single, viable and activated primary human T cells were transfected with C12-200 and DOTAP(C12-200) lipid nanoparticles (LNPs) encapsulating anti-CD20 nanoCAR mRNA (1 ng/µL and 3 ng/µL per 5 x 10^4^ T cells, respectively). 7-AAD Viability Staining solution (Biolegend, USA) was used to distinguish between live and dead cells. Staining of nanoCAR+ T cells following LNP transfection was performed with an iFluor 488-conjugated MonoRab Rabbit Anti-Camelid VHH (Genscript). Gating was performed on their respective mock controls, i.e., LNP-transfected T cells pre-treated with Human TruStain FcX™ (Biolegend).
